# 3D genome evolution and reorganization in the *Drosophila melanogaster* species group

**DOI:** 10.1371/journal.pgen.1009229

**Published:** 2020-12-07

**Authors:** Nicole S. Torosin, Aparna Anand, Tirupathi Rao Golla, Weihuan Cao, Christopher E. Ellison

**Affiliations:** Department of Genetics, Human Genetics Institute of New Jersey, Rutgers, The State University of New Jersey, Piscataway, New Jersey, United States; University of Wisconsin–Madison, UNITED STATES

## Abstract

Topologically associating domains, or TADs, are functional units that organize chromosomes into 3D structures of interacting chromatin. TADs play an important role in regulating gene expression by constraining enhancer-promoter contacts and there is evidence that deletion of TAD boundaries leads to aberrant expression of neighboring genes. While the mechanisms of TAD formation have been well-studied, current knowledge on the patterns of TAD evolution across species is limited. Due to the integral role TADs play in gene regulation, their structure and organization is expected to be conserved during evolution. However, more recent research suggests that TAD structures diverge relatively rapidly. We use Hi-C chromosome conformation capture to measure evolutionary conservation of whole TADs and TAD boundary elements between *D. melanogaster* and *D. triauraria*, two early-branching species from the *melanogaster* species group which diverged ∼15 million years ago. We find that the majority of TADs have been reorganized since the common ancestor of *D. melanogaster* and *D. triauraria*, via a combination of chromosomal rearrangements and gain/loss of TAD boundaries. TAD reorganization between these two species is associated with a localized effect on gene expression, near the site of disruption. By separating TADs into subtypes based on their chromatin state, we find that different subtypes are evolving under different evolutionary forces. TADs enriched for broadly expressed, transcriptionally active genes are evolving rapidly, potentially due to positive selection, whereas TADs enriched for developmentally-regulated genes remain conserved, presumably due to their importance in restricting gene-regulatory element interactions. These results provide novel insight into the evolutionary dynamics of TADs and help to reconcile contradictory reports related to the evolutionary conservation of TADs and whether changes in TAD structure affect gene expression.

## Author summary

Chromosomes are organized inside the three-dimensional nucleus in a hierarchical fashion, from the wrapping of DNA around nucleosomes to the presence of compartments of active and inactive chromatin. Here, we focus on domains of interacting chromatin known as topologically associating domains, or TADs. TADs are dozens to hundreds of kilobases in size and tend to contain genes with similar epigenetic states. While much research has been done on the mechanisms of TAD formation, less is known about the role of TADs in genome evolution. The research that does exist is contradictory, with some studies claiming that TADs are highly conserved and others reporting that TADs diverge rapidly. We compared the TAD locations between two Drosophila species and found that the majority have been reorganized. We also identified TAD subtypes that differ in terms of their evolutionary conservation: TADs that contain developmental genes are less likely to be reorganized while TADs that contain broadly expressed genes evolve rapidly. We found that TAD reorganization has an effect on gene expression, but only for genes located near the site of disruption. These results provide new insight into the evolution of 3D genome organization and help to reconcile previous contradictions in the literature.

## Introduction

The recent development of Hi-C sequencing techniques has allowed for inference of three-dimensional chromosome conformation through identification of inter- and intra-chromosomal interactions at high-resolution across the entire genome. Visualization of gene contacts and contact frequencies led to the discovery of organizational features called topologically associating domains, or TADs, which bring genes in close proximity with their regulatory elements [1]. TADs are regions of highly-interacting chromatin that contain genes with similar expression patterns and epigenetic states, and their location is conserved throughout development and across tissue types in both mammals and Drosophila [2–4]. Domains are demarcated by boundaries which are regions of decompacted chromatin bound by insulator proteins [5]. In vertebrates, the CCCTC-binding factor (CTCF), along with the structural maintenance of chromosomes (SMC) cohesin complex, play a major role in specifying TAD boundaries [2, 6–8], whereas in Drosophila, CTCF and SMC binding show little enrichment at TAD boundaries [9]. Instead, other insulator proteins, including BEAF-32, Chromator, CP190, and M1BP are more frequently found at TAD boundaries [3, 10–13] and depletion of M1BP has been shown to disrupt 3D genome organization in the Drosophila Kc167 cell line [10].

Most research thus far investigating 3D genome structure has operated under the prevailing theory that TADs regulate gene expression by limiting potential gene-enhancer interactions to those within a given domain. This theory is supported by a variety of studies in mammals. For example, genome-wide enhancer-promoter contacts in mouse neurons occur mainly within TADs [14] and reporter gene-enhancer interactions have been shown to be correlated with TAD structure [15]. Furthermore, disruption of TAD boundaries has been associated with aberrant enhancer/promoter contacts, gene misregulation, developmental abnormalities and cancer [16–21]. Developmental genes in Drosophila have also been shown to engage in long-distance interactions with distal regulatory sequences. Ghavi-helm et. al. [22] performed 4C-seq with viewpoints centered on known developmental enhancers and found that each enhancer interacted with two promoters and three other enhancers on average. Seventy-three percent of interactions spanned distances larger than 50 kb and the majority of interactions occurred within the same TAD. These long-distance interactions represent a level of 3D connectivity comparable to humans [22].

**Fig 1:**
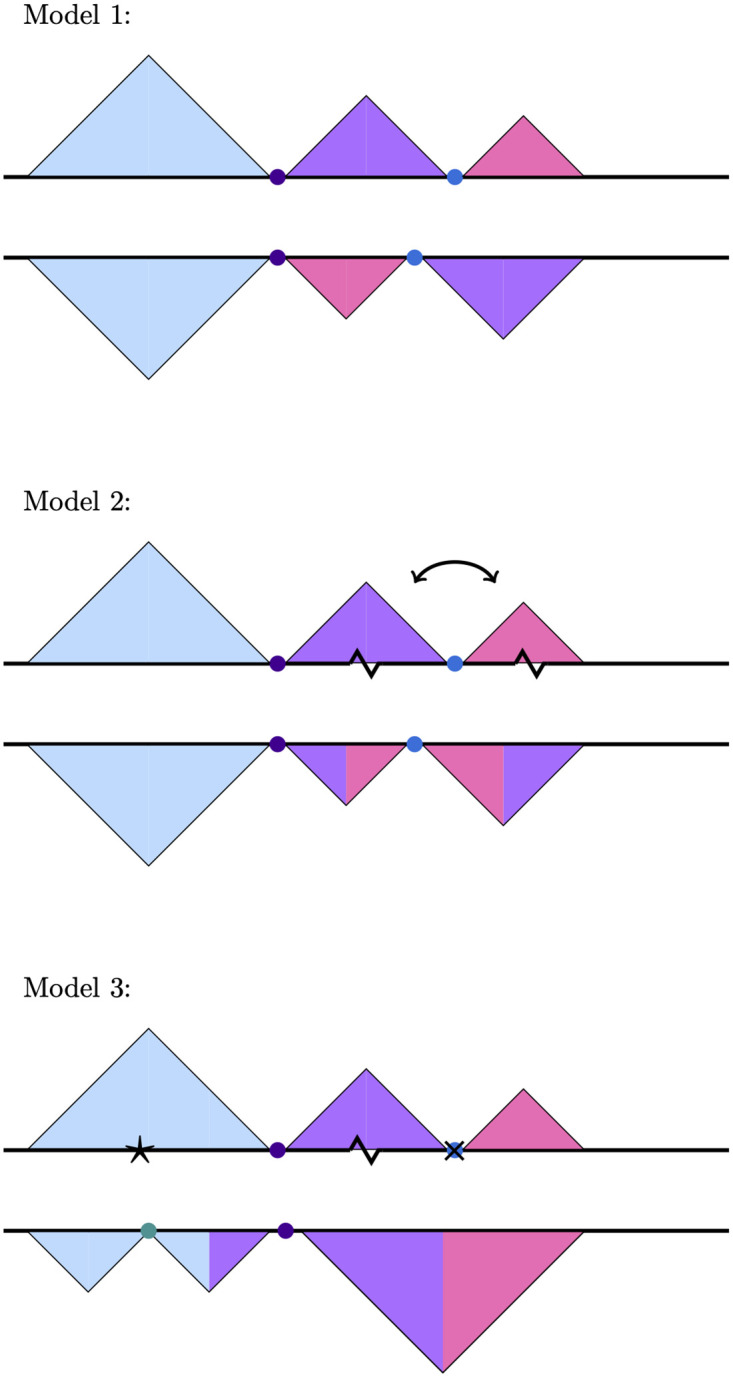
Models depicting possible TAD and boundary rearrangement scenarios. Each model shows TAD contact domains (triangles) and boundaries (circles) in two hypothetical species. Model 1: chromosomal rearrangements occur at TAD boundaries, resulting in domains shuffling as intact units, represented by the purple and lilac TADs. Model 2: TAD boundaries remain conserved but TADs themselves are disrupted. In this example, an inversion whose breakpoints (jagged lines) occur within two separate TADs results in TAD reorganization, seen here as the two chimeric purple/lilac TADs. Model 3: both TADs and their boundaries evolve rapidly. The gain and loss of boundary elements (black star and ‘X’, respectively), leads to further TAD reorganization, beyond that observed in Model 2.

The functional role of TADs with respect to the regulation of gene expression has important implications for 3D genome evolution. If TAD structure is critical for proper gene regulation, then the evolution of 3D genome organization should be highly constrained and related species should show strong conservation of TAD structures. Consistent with this prediction, a variety of studies in vertebrates have reported strong conservation of 3D genome organization using comparative Hi-C approaches [2, 7, 23, 24]. A recent study in Drosophila reported that 3D genome architecture is conserved over 40 million years of evolution in spite of extensive chromosomal rearrangements [25]. These studies support a model where chromosomal rearrangements that preserve TADs (i.e. their breakpoints are located within TAD boundaries) are much more likely to be retained over evolutionary time compared to rearrangements that disrupt TADs (Fig 1, Model 1). Under this model, TADs are shuffled as whole units over evolutionary time due to selection to maintain the 3D interaction properties of the genes and regulatory sequences within them.

However, more recent research suggests that TADs may be frequently reorganized over evolutionary time. Notably, one recent study found that only 43% of TADs are shared between humans and chimpanzees [26]. Furthermore, there are a number of studies showing that gene expression profiles remain unperturbed upon TAD reorganization. For example, the extensive changes in chromosome topology caused by the rearrangements found on a Drosophila balancer chromosome are not associated with widespread differences in gene expression [27]. Also in Drosophila, studies involving deletion mutations [28] and experimentally-induced inversions [29] found that these mutations, which should disrupt TAD organization, had little effect on gene expression. Similar observations have been made in mice: fusion of TADs does not have major effects on gene expression [30]. These studies suggest that TADs may diverge relatively rapidly over evolutionary time, with little effect on gene expression (see Models 2 and 3, Fig 1).

One possibility that might explain these apparently contradictory results is that there are distinct functional subtypes of TADs, with some being more tolerant of reorganization than others. Consistent with such a possibility, a recent study identified a subset of ancient, highly-conserved TADs in both vertebrates and flies that are enriched for conserved noncoding elements and developmental genes [31]. Based on findings that TAD locations and/or contact frequencies are conserved between species at a level beyond that expected by chance, previous studies in mammals [2, 7, 23] and flies [25] have concluded that TADs in general are evolving under strong constraint. However, different subtypes of TADs may also be subject to different evolutionary forces, with some, such as those containing developmental genes, evolving under purifying selection, while others could be evolving neutrally, or even under positive selection.

Here, we have compared 3D genome organization between *Drosophila melanogaster* and *Drosophila triauraria*, which diverged ∼15 million years ago [32]. We chose this species pair because, based on the evolutionary rate of chromosomal rearrangements inferred by [33], they should have accumulated extensive chromosomal rearrangements since their divergence, on the order of several hundred synteny breakpoints. We have improved a previously published *D. triauraria* genome assembly [34] by performing additional nanopore sequencing and Hi-C scaffolding which yielded chromosome-length scaffolds. We identified 991 rearrangement breakpoints between the *Drosophila melanogaster* and *Drosophila triauraria* genome assemblies, which contain blocks of synteny that are at least twice as large as TADs (∼120 Kb, see Results. Drosophila TADs: 23—63 Kb average length [10]). We then used two biological replicates of Hi-C sequencing data to identify high-confidence TADs and TAD boundaries in each species. Overall, we find that only 25% of TADs are orthologous between these two species. The majority of TADs have been reorganized by a combination of chromosomal rearrangements and TAD boundary gain/loss. Changes to TAD structures are associated with differential gene expression near the sites of disruption but not across entire TADs. We also find evidence that subtypes of TADs differ with respect to their evolutionary dynamics: conserved TADs are enriched for Polycomb-repressed and developmentally-regulated chromatin, while transcriptionally active TADs containing broadly-expressed genes evolve rapidly, possibly due to positive selection. On the other hand, TADs located in pericentric heterochromatin may be evolving neutrally. We propose that evolutionary divergence in 3D genome organization results from shuffling of conserved boundary elements across chromosomes along with the formation of lineage-specific boundaries. Both of these processes break old TADs and create new TAD architectures. Our results also support the existence of functionally distinct TAD subtypes: some TADs may be evolutionarily flexible and able to be reorganized without perturbing gene expression, whereas there may also be a distinct set of developmentally-regulated TADs that remain highly conserved due to their importance in restricting long-distance gene-regulatory element interactions.

## Results

### *D. triauraria* genome assembly

A recently published genome assembly for *D. triauraria* was made using relatively low-coverage (∼18.8x) nanopore sequencing data [34]. In order to create an improved assembly, we performed additional long-read nanopore sequencing of genomic DNA extracted from ∼30 adult females from *D. triauraria* strain 14028-0651.00 (National Drosophila Species Stock Center at Cornell). We used three r9.4 flow cells to generate a total of 633,844 reads (10,287 bp mean length) and 6.5 Gb of sequencing data. We combined our data with the previously published nanopore data from *D. triauraria* [34] for a final dataset of 1,043,600 reads (total size: 10.5 Gb, coverage: 52x). We basecalled the raw signal data with *Albacore* (version 2.3.4, available from Oxford Nanopore Technologies) and assembled the basecalled reads with *Canu* [35], which produced an assembly with contig N50 of 1.3 Mb (1098 total contigs, 269 Mb total size). We then polished the assembly by using *Nanopolish* [36] with raw nanopore signal data and *Pilon* [37] with Illumina data, which corrected a total of 1,185,510 assembly errors. Next, we used *Purge Haplotigs* [38] to identify allelic contigs, where highly heterozygous haplotypes were assembled as separate contigs rather than collapsed. After removing secondary haplotigs and bacterial contigs, our final contig assembly consisted of a total of 294 contigs which sum to ∼200 Mb and have an N50 of 1.7 Mb, which is 2.4X larger than the previously published study (N50 = 720 Kb) [34] (S1 Table).

**Fig 2:**
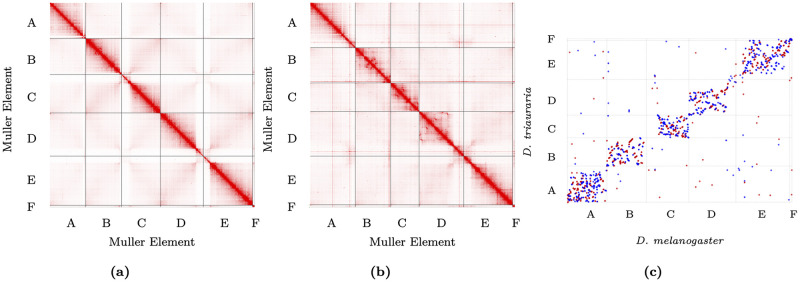
Hi-C contact maps and genome comparison. A) *D. melanogaster* contact map; B) *D. triauraria* contact map. Contact maps show frequencies of pairwise 3D contacts, inferred from Hi-C data. Darker colors represent higher contact frequencies. Contact frequencies were visualized using *Juicebox* [39]. C) *MUMmer* dotplot depicting chromosomal rearrangements between *D. melanogaster* and *D. triauraria*. The promer utility from *MUMmer* [47] was used to visualize synteny between *D. melanogaster* and *D. triauraria*. Each dot corresponds to a one-to-one alignment between the two genomes. Red dots represent + /+ strand alignments and blue dots represent +/− strand alignments.

We next performed Hi-C scaffolding using the polished nanopore contigs and the software packages *Juicer* [39] and *3D-DNA* [40] (Fig 2A and 2B). The scaffolding process produced chromosome-length scaffolds, reflected by the dramatic increase in N50 from 1.7 Mb to 34.5 Mb. The gene content of chromosome arms is conserved across Drosophila, and all Drosophila species contain five chromosome arms, which are designated as Muller Elements A-F. In order to assign the *D. triauraria* scaffolds to Muller elements, we performed a translated BLAST search of our scaffolds using *D. melanogaster* peptides as queries and keeping only the best hit for each query sequence. We found that each scaffold was highly enriched for *D. melanogaster* peptides from a specific Muller element (S1 Fig) and we successfully identified scaffolds corresponding to Muller elements A-F for downstream analysis. In order to predict complete gene models for our assembly, we generated RNA-seq data from *D. triauraria* ovaries, testes, and embryos. We combined the RNA-seq data with *ab initio* gene predictions from *Augustus* [41] and *SNAP* [42] and homology-based predictions from *D. melanogaster* peptides using *MAKER* [43].

*D. triauraria* was previously sequenced unintentionally because it was mislabeled as *D. kikkawai* [34], which means that the published *D. triauraria* nanopore data are from an unknown strain. The strain we sequenced is from the same stock center, making it likely that the contaminant and our strain, are in fact the same strains. To test this, we aligned the Illumina data from Miller et. al. [34], in conjunction with our uninformative Hi-C reads, to our nanopore assembly and called SNPs using *FreeBayes* [44]. We compared the genotypes from the Miller et. al. [34] strain to our strain at ∼93.7 million sites and found that 93.5 million sites (∼99.8%) were homozygous reference in both datasets, while at 215,000 sites, both strains had the same heterozygous genotype. For another 37,000 sites, one strain was identified as homozygous and the other heterozygous. The strains were in complete disagreement (i.e. they were homozygous for different alleles) at only 3 sites. From this analysis, we concluded that the *D. triauraria* strain mislabeled *D. kikkawai* was in fact the same strain we sequenced.

Several nested polymorphic inversions have been found to be segregating at high frequencies in *D. triauraria*. Mavragani et. al. [45] identified a set of nested inversions on two different chromosomes in *D. triauraria* based on inspection of polytene chromosomes but did not assign these chromosomes to Muller elements. We manually inspected the Hi-C contact map for evidence of polymorphic inversions and identified two inversions on Muller B, two inversions on Muller C, and three inversions on Muller D (S2 Fig).

### Genome synteny

We next sought to identify synteny blocks and assess the degree of chromosomal rearrangements between *D. melanogaster* and *D. triauraria*. We created an orthology map between the genome assemblies for these two species using *Mercator* [46] and identified a total of 991 synteny blocks with average size of ∼117 Kb in *D. melanogaster* and ∼140 Kb in *D. triauraria*. The larger size of the synteny blocks in *D. triauraria* is consistent with the larger genome size for this species. We visualized synteny by using the promer tool from the *MUMmer* pipeline [47] to produce a dotplot (Fig 2C), which shows that there have been extensive chromosomal rearrangements since the divergence of these two species, with the majority of rearrangements occurring within Muller elements, as has been found for other Drosophila species [48].

Errors in the genome assembly would affect our synteny analysis because misassembled regions would be interpreted as chromosomal rearrangements. To assess our confidence that the *D. triauraria* genomic regions that overlap synteny breakpoints are assembled correctly, we aligned the raw nanopore reads back to the assembly and identified reads that spanned synteny breaks by at least 1 kb on either side. If the synteny breaks are due to misassembly, there should be few, if any, raw sequencing reads that span these breaks. Instead, we find that each synteny breakpoint is spanned by an average of 23 nanopore reads, which is similar to the coverage of randomly selected genomic regions (shuffled breakpoint average: 22.95). Only 0.4% of breakpoints (4 out of 963) are spanned by less than 5 reads (S3 Fig).

### TAD boundary and domain annotation

In order to determine how the large number of chromosomal rearrangements present between these two species has affected 3D genome organization, we identified TAD boundaries as well as complete contact domains (i.e. TADs) in both species. We used *HiCExplorer* [10], which was developed using Drosophila Hi-C data, to identify TAD boundaries at 5 kb resolution for both *D. melanogaster* and *D. triauraria*. The total number of Hi-C read pairs for each dataset are reported in S2 Table. *HiCExplorer* calculates the TAD separation score for each bin in the genome and identifies TAD boundaries as those bins whose score shows significantly larger contact insulation compared to neighboring bins. We used a bin size of 5 kb and found that the TAD separation scores were highly correlated between replicate datasets for each species (Spearman’s rho: 0.995 [*D. melanogaster*] and 0.990 [*D. triauraria*] (S4(A) and S4(B) Fig). We also found that the majority of predicted boundaries were identified in each replicate independently (74% [*D. melanogaster*], 70% [*D. triauraria*]). We refer to the boundaries that were identified in both replicates as high confidence boundaries, and those identified in only one of the two replicates as low confidence boundaries. In total, we identified 701 and 843 high confidence TAD boundaries for *D. melanogaster* and *D. triauraria*, respectively, and 249 and 355 low confidence boundaries (S3 Table).

*HiCExplorer* [10] links TAD inter-boundary regions together into contact domains. Similar to our approach with boundary elements, we identified contact domains that were found independently in both replicate datasets as high confidence domains and those found only in one replicate as low confidence domains. In total, we identified 552 and 639 high confidence TAD domains for *D. melanogaster* and *D. triauraria*, respectively, and 593 and 811 low confidence domains (S3 Table).

### Boundary motif enrichment

In Drosophila, TAD boundaries are highly enriched for motifs recognized by the insulator proteins M1BP and BEAF-32 [10]. To validate boundary calls made by *HiCExplorer* [10], we used *Homer* [49] software to search the identified boundaries for enriched sequence motifs. Boundaries from both species were enriched for motifs recognized by M1BP (*p* = 1e–17 [*D. melanogaster*], *p* = 1e–42 [*D. triauraria*]) and BEAF-32 (*p* = 1e–18 [*D. melanogaster*], *p* = 1e–15 [*D. triauraria*]), which supports the accuracy of our boundary calls (S5 Fig).

### Domain and boundary conservation

We assessed the evolutionary conservation of TAD boundaries between *D. melanogaster* and *D. triauraria* by lifting over the high confidence *D. melanogaster* boundary coordinates to the *D. triauraria* genome coordinates. We created a whole-genome alignment between the two genome assemblies using *Cactus* [50] and performed the coordinate liftovers using the *halLiftover* [51] utility. We considered boundaries to be orthologous when high confidence boundary regions lifted-over from *D. melanogaster* to *D. triauraria* overlapped either a high or low confidence boundary that was independently identified in *D. triauraria*. Out of a total of 701 boundaries identified in *D. melanogaster*, 654 were successfully lifted-over to a corresponding region in *D. triauraria*. Of the lifted-over boundaries, 473 (∼72%) are orthologous between the two species and 181 (∼28%) are melanogaster-specific (Table 1). Our results suggest that the majority of TAD boundaries have been conserved since the divergence of these two species ∼15 Mya.

**Table 1:** Summary of results from *D. melanogaster* to *D. triauraria* liftover analysis. Percentages of “Unique lifted-over to *D. triauraria*” represent number out of total boundaries or domains. Percentages of orthologous, non-orthologous, and subcategories of boundaries and domains represent number out of “Unique lifted-over to *D. triauraria*”. Truncated and expanded domains do not meet the 90% reciprocal overlap criterion due to large insertion/deletion mutations that have created asymmetry in TAD size between the two species. Missing boundaries and domains are those that could not be lifted-over to *D. triauraria*.

Category	Boundaries	Domains
Total in *D. melanogaster*	701	552
Unique lifted-over to *D. triauraria*	654 (93%)	544 (99%)
Orthologous	473 (72%)	134 (25%)
Non-orthologous	181 (28%)	410 (75%)
Non-orthologous (truncated/expanded)	–	82 (15%)
Non-orthologous (split by lineage-specific boundary)	–	104 (19%)
Non-orthologous (split by rearrangement)	–	224 (41%)
Missing	47	8

We next sought to determine whether TADs themselves showed a similar degree of conservation in spite of the large number of chromosomal rearrangements between these species. TADs would remain conserved if chromosomal rearrangements occur in such a way that TADs are shuffled as intact units (see Model 1, Fig 1). Alternatively, it is possible that the sequence motifs that specify boundaries remain conserved while chromosomal rearrangements shuffle these sequence elements in ways that lead to widespread TAD reorganization (see Model 2, Fig 1). Finally, both chromosomal rearrangements and boundary gain and loss could contribute to TAD evolution (see Model 3, Fig 1). To differentiate between these possibilities, we identified orthologous contact domains between these two species. Similar to our approach with boundaries, we considered contact domains to be orthologous when high confidence domain regions from *D. melanogaster* lifted-over as a continuous block (allowing for internal rearrangements) to *D. triauraria* and overlapped either a high or low confidence TAD domain that was independently identified in *D. triauraria*. We required that the domains were reciprocally overlapping by at least 90% of their lengths.

**Fig 3:**
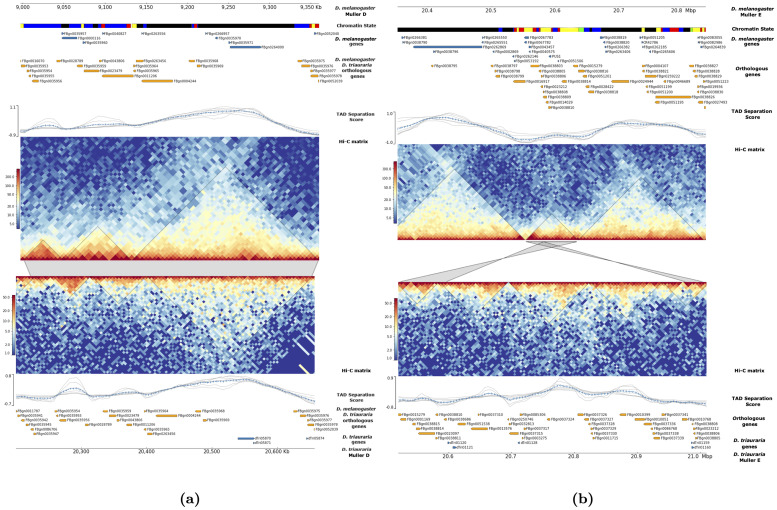
Visualization of orthologous and non-orthologous TADs. A) Three orthologous, conserved TADs on Muller D between *D. melanogaster* (upper heatmap) and *D. triauraria* (lower heatmap). B) Non-orthologous, split TAD on Muller E between *D. melanogaster* (upper heatmap) and *D. triauraria* (lower heatmap). The black triangles on the contact matrices show the locations of TADs. The chromatin state annotations are based on Filion et. al. [53]. The chromatin classifications are as follows: BLACK: inactive, BLUE: Polycomb-repressed heterochromatin, GREEN: constitutive heterochromatin, RED: dynamic active, YELLOW: constitutive active. Orthologous genes are labeled by their FlyBase IDs and Hi-C matrices were generated by *HiCExplorer*. The grey blocks connecting matrices indicate syntenic regions. In B) gene tracks show that genes such as FBgb0038805, FBgn0038806, and FBgn0038814 are split between different TADs in *D. triauraria* and are in the reverse orientation compared to *D. melanogaster*.

Out of a total of 552 domains identified in *D. melanogaster*, 544 were successfully lifted-over to a corresponding region in *D. triauraria*. Of the lifted-over domains, we found that 134 (25%) are orthologous between the two species, whereas 410 (75%) of the *D. melanogaster* TADs do not show a one-to-one relationship with a *D. triauraria* TAD (Table 1). Of the non-orthologous TADs, 41% (224/544) (Table 1), are due to cases where TADs have been split by chromosomal rearrangements (i.e. a contiguous *D. melanogaster* domain lifts over to multiple, discontiguous regions in *D. triauraria*) (Fig 3B). Of the orthologous domains, 84 (∼63%) also shared orthologous boundary regions.

The Drosophila X chromosome has previously been shown to accumulate chromosomal rearrangements at a faster rate compared to the autosomes [33]. We found a similar pattern for *D. melanogaster* and *D. triauraria*, where the median size of synteny blocks is significantly lower for the X chromosome compared to the autosomes (S6 Fig, Wilcoxon test *p* = 1.35e–12). We also found that the proportion of orthologous TADs on the X chromosome is reduced relative to the autosomes (S6 Fig, Fisher’s Exact Test *p* = 0.014), consistent with increased structural divergence of the X chromosome leading to increased TAD reorganization.

The large disparity between the fraction of orthologous boundaries versus orthologous TADs could simply be due to the fact that TADs present a much larger mutational target and will therefore be more likely to contain a rearrangement breakpoint compared to the boundary regions. To determine whether this was the case, we classified TADs and boundaries based on whether they are located in genomic regions that are co-linear between the two species versus regions that have been interrupted by a chromosomal rearrangement. We found that 42% of TADs and 84% of TAD boundaries are co-linear, which is very similar to the numbers expected by chance (TADs: 230 versus 238 expected, Boundaries: 586 versus 587 expected). However, co-linear genomic regions will not contain orthologous TADs if there are species-specific TAD boundaries in these regions. Boundaries could be gained or lost in co-linear regions via short indel or point mutations that create or remove insulator binding sites while still maintaining homology between the two species. Indeed, we find that slightly more than half (58%) of TADs within co-linear genomic regions are orthologous (defined by reciprocal overlap of at least 90% of their length) while the co-linear TADs that are non-orthologous have either been altered by lineage-specific TAD boundaries or contain enough insertions/deletions that they do not meet the 90% reciprocal overlap criterion for orthology (Table 1).

Given that only 25% of domains are orthologous between the two species, we conclude that both chromosomal rearrangements and boundary gain/loss have reorganized the majority of TADs present in each of these species since their common ancestor, making Model 3 (Fig 1) the most likely scenario for TAD evolution. For consistency, we repeated these analyses by performing the liftover in the opposite direction, from *D. triauraria* to *D. melanogaster*, and obtained similar results (S4 Table).

### Gene expression

**Fig 4:**
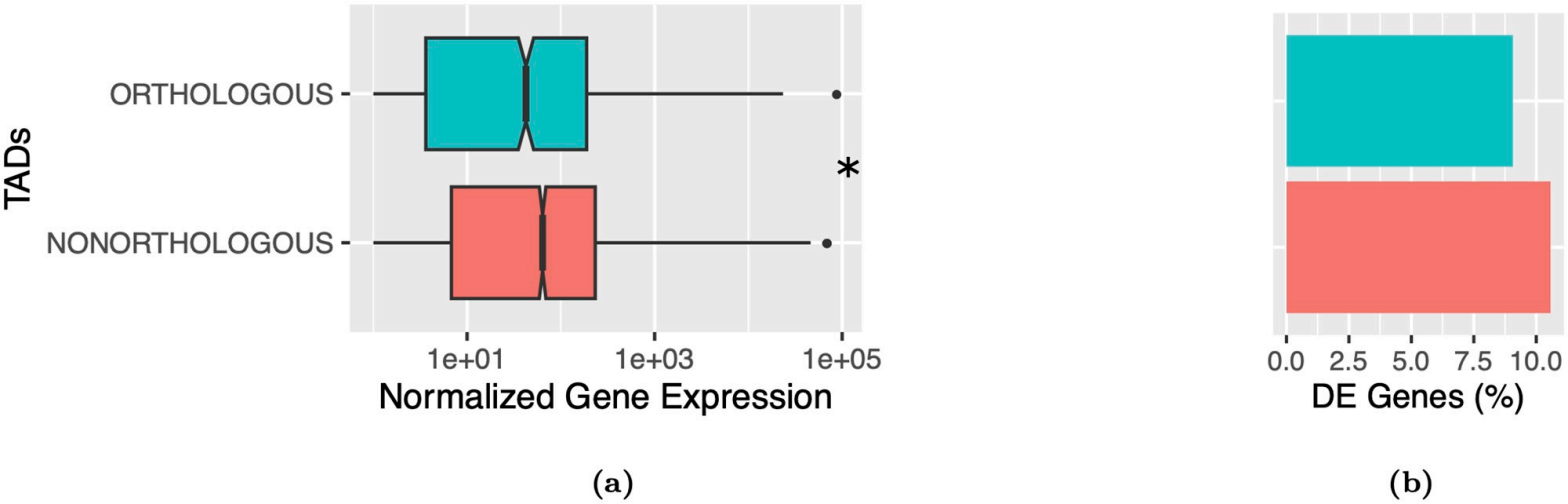
Gene expression in orthologous versus non-orthologous TADs. A) Genes within orthologous TADs are expressed at significantly reduced levels compared to non-orthologous TADs, consistent with Polycomb-repression (Wilcoxon test *p* = 6.7e–05) B) Orthologous TADs contain slightly fewer differentially-expressed (DE) genes compared to non-orthologous TADs (9.1% versus 10.5%), however this difference is not statistically significant (Fisher’s Exact Test *p* = 0.151). Differentially-expressed genes were identified using the *DESeq2* R software package [52].

**Fig 5:**
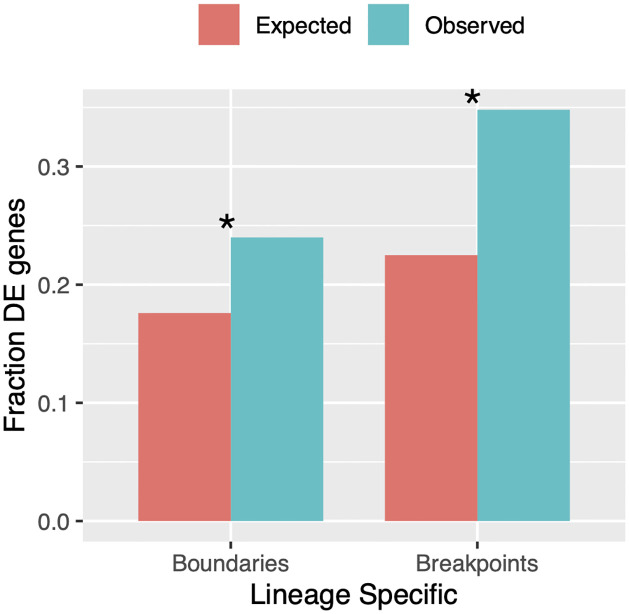
Differentially-expressed genes are enriched near locations where TADs are disrupted. We calculated the fraction of differentially-expressed genes (DE) that lie within 10 kb of locations where TADs have been disrupted via chromosomal rearrangements or lineage-specific TAD boundaries. In both cases, we found significantly more differentially-expressed genes than expected by chance: Lineage-specific boundaries: observed: 24.0% [231], expected: 17.6% [170], hypergeometric *p* = 7.9e–8. Rearrangement breakpoints: observed: 34.8% [335], expected 22.5% [217], hypergeometric *p* = 2.6e–23.

We hypothesized that TADs rearranged in *D. triauraria* compared to *D. melanogaster* might reorganize enhancer-promoter contacts and result in altered gene expression profiles. We performed RNA-seq on replicate datasets for each species and used the *DESeq* R package [52] to identify differentially-expressed genes between the two species. A total of 964 differentially-expressed genes were identified (S7 Fig). We then compared the expression of genes within orthologous and non-orthologous TAD domains between the two species and found that, while nonconserved TADs show a slightly higher percentage of differentially-expressed genes (10.5% versus 9.1%), this difference is not significant (Fig 4, Fisher’s Exact Test *p* = 0.151). These findings suggest that TAD reorganization in Drosophila does not result in widespread changes in gene expression. To determine if changes in TAD structures exert a more localized effect near the site of disruption, we examined differentially-expressed genes within 10 kb of rearrangement breakpoints and lineage-specific TAD boundaries. The fraction of differentially-expressed genes within 10 kb of lineage-specific boundaries is significantly larger than expected by chance (observed: 24.0% [231], expected: 17.6% [170], hypergeometric *p* = 7.9e–8). We observed a similar pattern for rearrangement breakpoints, after excluding breakpoints that overlapped TAD boundaries (Fig 5, observed: 34.8% [335], expected: 22.5% [217], hypergeometric *p* = 2.6e–23).

There is evidence that the act of transcription itself plays a role in TAD boundary formation [13] which raises the possibility that the association between non-orthologous boundaries and differentially-expressed genes is due to changes in gene expression in *cis*, rather than changes in TAD organization. For example, mutations in promoters and/or transcription factor binding sites could cause downregulation of genes near a TAD boundary, which could then cause the boundary itself to weaken or disappear. Under this scenario, differentially-expressed genes near lineage-specific TAD boundaries should show increased expression in the species where the boundary is present and reduced expression in the species where the boundary is absent. We examined *D. melanogaster* lineage-specific TAD boundaries to determine whether there is support for this prediction. We find that there is no difference between the percent of upregulated genes within 10 kb of lineage-specific versus orthologous TAD boundaries (54.5% versus 53.7%, Fisher’s Exact Test *p* = 0.49). These results suggest that differences in transcription are not driving differences in boundary presence/absence.

**Fig 6:**
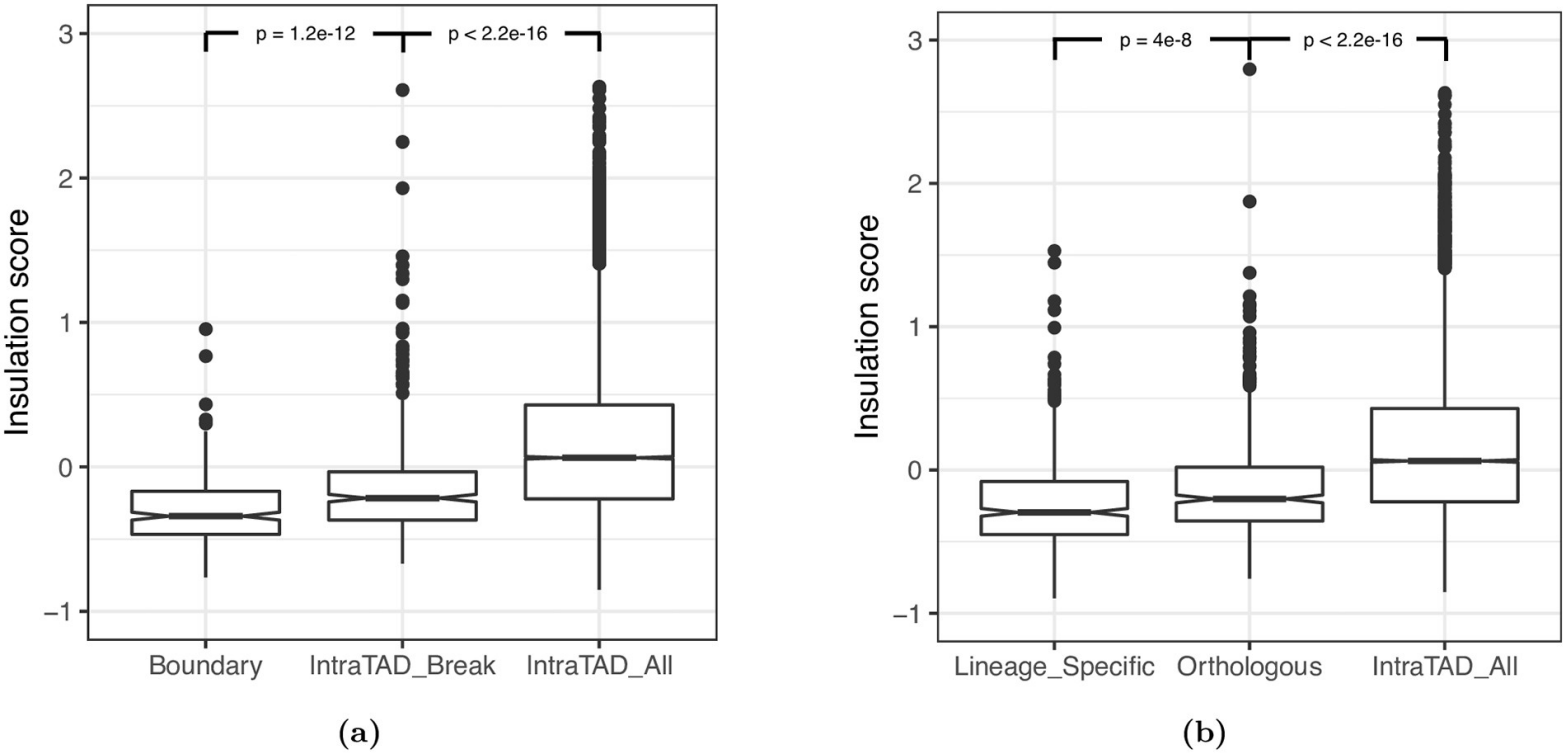
TAD disruptions tend to occur at insulator-like regions. We analyzed *HiCExplorer* insulation scores calculated for 5 kb bins across the *D. melanogaster* and *D. triauraria* genomes. A) We found that intra-TAD breakpoints tend to occur at genomic regions that have increased insulation relative to other intra-TAD bins: Wilcoxon test *p* < 2.2e–16, but significantly less insulation compared to TAD boundaries: Wilcoxon test *p* = 1.2e–12. B) We also examined lineage-specific boundaries and found that the genomic regions orthologous to lineage-specific boundaries show increased insulation relative to all intra-TAD 5 kb bins: Wilcoxon test *p* < 2.2e–16. However, lineage-specific boundaries have significantly increased insulation compared to the orthologous region in the other species, supporting their classification as lineage-specific: Wilcoxon test *p* = 4e–8. Note that more negative scores indicate more insulation. Full plot for B in S9 Fig.

Even near the endpoints of disrupted TADs, the vast majority of genes (∼87%) are expressed at similar levels between the two species, which suggests that the effects of TAD reorganization on gene expression is relatively subtle. To investigate why this would be the case, we compared insulation scores for rearrangement breakpoints located within 5 kb of a TAD boundary to intra-TAD breakpoints located more than 5kb from TAD boundaries as well as the insulation scores of all intra-TAD 5 kb bins. We found that intra-TAD breakpoints tend to occur at regions with increased insulation, compared to all intra-TAD bins (Wilcoxon test *p* <2.2e–16), but significantly less insulation compared to TAD boundaries (Fig 6A, Wilcoxon test *p* = 1.2e–12). We additionally examined lineage-specific boundaries to determine whether novel boundaries tend to evolve at genomic regions with pre-existing insulation activity. We found that the orthologs of lineage-specific boundaries show increased insulation relative to all intra-TAD 5 kb bins (Fig 6B, Wilcoxon test *p* < 2.2e–16), consistent with a tendency for boundaries to emerge from genomic regions with pre-existing insulating properties. Additionally, we found that compared to the orthologous region in the other species, lineage-specific boundaries have significantly increased insulation (Wilcoxon test *p* = 4e–8) and significantly more insulator protein (BEAF-32 and M1BP) binding motifs (S5 Table, paired Wilcoxon test *p* = 0.0059), supporting their classification as lineage-specific, and implying that lineage-specific boundaries evolve via the accumulation of insulator protein binding motifs. From these analyses, we conclude that TAD reorganization is associated with changes in gene expression for a subset of genes located near the site of disruption. The subtle effect of TAD reorganization on gene expression may be due, at least in part, to the fact that both rearrangements and lineage-specific boundaries tend to occur at locations that had insulating properties in the common ancestor of the two species.

### Chromatin state

**Fig 7:**
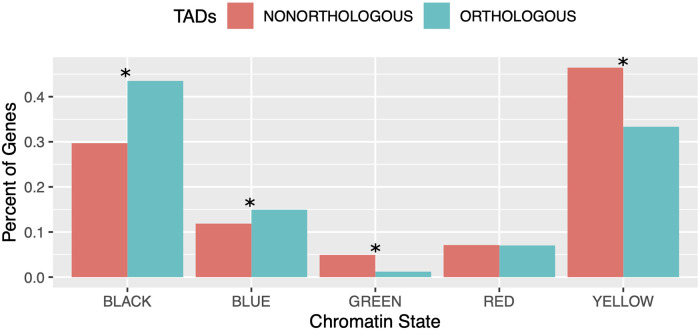
Orthologous TADs are enriched for transcriptionally silent and Polycomb-repressed genes. The bar plot shows the percent of genes in each chromatin state (defined by [53]) in orthologous versus non-orthologous TADs. Orthologous TADs are significantly enriched for the BLACK (inactive) and BLUE (Polycomb-repressed) chromatin states and significantly depleted of the GREEN and YELLOW chromatin states. Asterices * indicate significant differences as calculated by Fisher’s Exact Test (p-values: BLACK = 1.225e–25; BLUE = 4.322e–4; GREEN = 1.552e–15; YELLOW = 8.375e–23; RED = 0.398).

Given that TAD locations are correlated with the epigenetic state of chromatin, we next sought to determine whether the properties of TADs differ depending on their chromatin state. We first compared chromatin states between genes in orthologous and non-orthologous TADs. We quantified the number of genes in each of the five chromatin states described by Filion et al. [53] within orthologous and non-orthologous TAD regions (S6 Table). Orthologous TADs show significant enrichment of the BLACK (transcriptionally silent) and BLUE (Polycomb-repressed) chromatin states and significant depletion of the GREEN (constitutive heterochromatin) and YELLOW (constitutively active) chromatin states, compared to non-orthologous TADs (Fig 7, Fisher’s Exact Test *p*s: 1.225e–25 [BLACK], 4.322e–4 [BLUE], 1.552e–15 [GREEN], 8.375e–23 [YELLOW]).

Polycomb-repressed chromatin is bound by Polycomb-group (PcG) proteins which regulate the epigenetic silencing of developmental genes. The BLACK chromatin state also contains developmentally-regulated genes [53]. It is associated with genes showing high tissue-specificity and contains a high density of conserved non-coding elements [53]. Consistent with orthologous TADs being enriched for epigenetically silenced developmental genes, we found that genes in orthologous TADs are expressed at significantly lower levels than those in non-orthologous TADs (Fig 4A, Wilcoxon test *p* = 6.7e–05). We also found that orthologous TADs are enriched for homeobox domain-containing genes (FlyMine protein domain enrichment test, Benjamini-Hochberg corrected *p* = 0.02) [54] and, in comparison to the genes within non-orthologous TADs, are also highly enriched for genes predicted to be regulated by Polycomb-group proteins (Fisher’s Exact Test *p* = 2.6e–5) [55]. The chromatin state tracks in Fig 3A and 3B also support our findings. The majority of genes in the conserved TADs in Fig 3A are BLACK and BLUE, while the genes within the split TAD in Fig 3B are predominantly YELLOW. These results largely mirror the chromatin states of the ancient and highly-conserved contact domains identified by Harmston et. al. [31], which contain clusters of conserved non-coding elements and developmental genes.

We next examined the chromatin states of chromosomal rearrangement breakpoints that disrupt TADs. If chromosomal rearrangements evolve neutrally, the chromatin states of polymorphic rearrangement breakpoints should show the same relative abundance as the chromatin states of breakpoints that differ between species, since both will be determined primarily by mutation rate [56]. Divergence between the chromatin state locations of polymorphic versus fixed breakpoints is likely to be due to the effects of natural selection. For example, chromatin states where gene order is under strong purifying selection should show a paucity of interspecies breakpoints whereas interspecies breakpoints should be elevated in chromatin states that tend to contain beneficial rearrangements.

**Fig 8:**
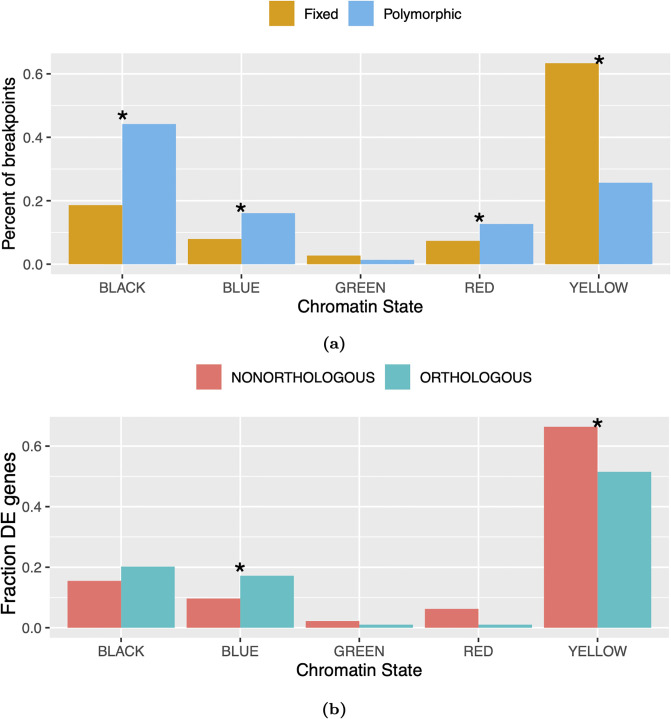
Evolutionary constraint of TADs by chromatin state. A) To assess whether TAD subtypes are under different levels of evolutionary constraint, we compared the frequency of polymorphic versus fixed intra-TAD rearrangement breakpoints across chromatin states. We found that interspecies rearrangement breakpoints that disrupt TADs are significantly depleted from the BLACK, BLUE, and RED chromatin states, and significantly enriched in the YELLOW chromatin state. These results suggest that rearrangement breakpoints from BLACK, BLUE, and RED states are under purifying selection, while some of the rearrangements in YELLOW states may have been fixed due to positive selection. Asterices * indicate significant differences as calculated by Fisher’s Exact Test (p-values: BLACK = 5.118e–17; BLUE = 9.714e–5; GREEN = 0.136; YELLOW = 2.352e–29; RED = 0.005). B) Y-axis represents fraction of differentially-expressed (DE) genes assigned to each chromatin state. DE genes from the YELLOW chromatin state are more likely to be located in disrupted (i.e. nonorthologous) TADs compared to conserved (i.e. orthologous) TADs, whereas the opposite is true for genes from the BLUE chromatin state. Asterices * indicate significant differences as calculated by Fisher’s Exact Test (p-values: BLACK = 0.156; BLUE = 0.026; GREEN = 0.381; YELLOW = 0.004; RED = 0.123).

We compared the chromatin states of polymorphic rearrangement breakpoints identified from long-read sequencing of 14 *D. melanogaster* strains [57] to the chromatin states of intra-TAD rearrangement breakpoints between *D. melanogaster* and *D. triauraria*. The interspecies rearrangement breakpoints are significantly depleted from the BLACK, BLUE, and RED chromatin states, relative to polymorphic breakpoints, whereas there is a large excess of interspecies breakpoints in the YELLOW chromatin state (active euchromatin) (Fig 8A). We also found that, specifically for the YELLOW chromatin state, a higher fraction of genes in non-orthologous TADs are differentially expressed, compared to orthologous TADs (Fig 8B, Fisher’s Exact Test *p* = 0.0059). These results show that interspecies rearrangement breakpoints that disrupt TADs are highly enriched in the YELLOW chromatin state and these disrupted TADs are associated with increased divergence in gene expression.

Genes in the YELLOW chromatin state tend to be broadly expressed across many tissues. To determine whether it is reasonable that this category of genes would be associated with adaptive changes in gene expression, we examined the chromatin states of genes whose divergence in expression level across seven species of Drosophila was previously identified as being due to positive selection [58]. We found that genes from the YELLOW chromatin state are highly enriched in this gene set (observed: 57.5%, expected: 40.1%, Fisher’s Exact Test *p* < 2.2e-16).

An inconsistency in our findings is that intra-TAD rearrangement breakpoints are depleted from the RED chromatin state, similar to the BLUE and BLACK states, but orthologous TADs are not enriched for RED genes (Fig 7), as they are for BLUE and BLACK genes. One explanation for this pattern is that TADs containing RED genes are less likely to be disrupted by chromosomal rearrangements but more likely to be split by lineage-specific boundaries. Consistent with this pattern, we find that lineage-specific boundaries are significantly closer to RED genes than expected by chance (S8 Fig, permutation *p* < 0.001).

These findings are consistent with a subset of interspecies rearrangements resulting in adaptive changes in gene expression associated with TAD reorganization. On the other hand, the depletion of breakpoints in the BLACK and BLUE chromatin states suggests that such rearrangements may be under purifying selection. The GREEN chromatin state, which is associated with constitutive heterochromatin, shows no difference in frequency between polymorphic and interspecies breakpoints, consistent with neutral evolution.

## Discussion

In this study, we sought to examine the evolutionary conservation of 3D genome organization in Drosophila. We selected *D. melanogaster* and *D. triauraria* for this comparison because they are separated by ∼15 million years of evolution [32]. We predicted that this level of divergence would be long enough that large-scale chromosomal rearrangements would have occurred between the two species but short enough that conservation at the nucleotide level would allow for an accurate whole-genome alignment. We used a combination of nanopore and Illumina Hi-C sequencing data to improve a recently published *D. triauraria* genome assembly produced from relatively low-coverage (depth 18.8x) nanopore sequencing data [34]. We have previously shown that Hi-C data can be used to scaffold Drosophila nanopore contigs with high accuracy, and even correct contig misassemblies [59]. We used our Hi-C data to scaffold the *D. triauraria* nanopore contigs and our improved *D. triauraria* assembly resulted in chromosome-length scaffolds highly enriched for genes corresponding to a single Muller element (S1 Fig), further supporting the efficacy of this approach. We were able to align ∼87% of our *D. triauraria* assembly to the *D. melanogaster* reference assembly and we found extensive chromosomal rearrangements (Fig 2C), consistent with our initial prediction that *D. triauraria* and *D. melanogaster* represent an ideal species pair for use in a comparative study of 3D genome organization.

Previous research has yielded conflicting results regarding the evolutionary conservation of TAD domains. In theory, TADs should be under strong purifying selection due to their role in preventing aberrant gene-enhancer interactions. Therefore, we expected that entire TAD contact domains, including boundary regions, would be conserved (i.e. Model 1, Fig 1). However, we found that the majority of TADs between *D. triauraria* and *D. melanogaster* are non-orthologous due to a combination of boundary elements being shuffled by chromosomal rearrangements and gain/loss of lineage-specific boundaries resulting in reorganization of TAD architecture (see Model 3, Fig 1). Our approach is conservative and likely underestimates the true extent of TAD divergence. Previous studies have identified inconsistencies in TAD-calling software packages [60] and have raised the possibility that TAD conservation results may depend on the direction of the liftover comparison [26]. For example, some studies report conservation estimates by first calling TADs in the species for which they have more data and then identifying the orthologous domains in the species for which they have less data [2, 6, 25, 26]. When reversing the analysis the conservation rate can be reduced by up to 25% [26]. However, in this study, we used biological replicates to demonstrate that the identification of TAD boundaries and TAD units is highly reproducible. We also performed our analysis of TAD conservation in both directions (i.e. from *D. melanogaster* to *D. triauraria* and vice versa) and obtained similar results regardless of the direction of comparison. Furthermore, our estimates of conservation, if biased at all, should be biased towards inferring higher levels of conservation. We only considered TADs for our liftover step if they were independently identified in both biological replicates, which should enrich for stronger TADs. After liftover, we considered the TAD to be orthologous if it overlapped *either* a strong (i.e. high-confidence) TAD *or* a weak TAD (i.e. low-confidence TAD identified in only a single replicate). We also did not require orthologous TADs to have orthologous boundaries. Instead, they were only required to have a reciprocal overlap of at least 90% of their lengths. We would expect these relatively low-stringency criteria to potentially result in an over-estimate of TAD conservation, yet we still only find ∼25% of TADs to be orthologous between species.

Broadly, our results are similar to several recent studies suggesting that TADs may actually diverge relatively rapidly and that TAD reorganization is not necessarily associated with widespread divergence in gene expression [26–30]. However, although non-orthologous TADs are not enriched for differentially-expressed genes (Fig 4B), we do find evidence for a localized effect of TAD reorganization on the expression of genes near the point of disruption, for both chromosomal rearrangements and lineage-specific TAD boundaries (Fig 5). This is similar to the effect reported by Ghavi-Helm et. al. [27] where disrupted TADs in the highly-rearranged balancer chromosomes of *D. melanogaster* showed more differences in gene expression near rearrangement breakpoints, rather than widespread across the entire TAD. Even near the sites of TAD disruption, the vast majority of genes are expressed similarly, both in the balancer chromosome study and in our comparison between *D. triauraria* and *D. melanogaster*. Interestingly, we find that both intra-TAD rearrangements and lineage-specific boundaries tend to occur at genomic regions that had insulator-like properties in the ancestor of these two species (Fig 6). These results raise the possibility that physical contacts spanning such regions were limited in the ancestral TAD configuration, which would explain the relatively subtle effect on gene expression that accompanied reorganization of these TADs.

Our results also provide insight into the evolution of TAD boundaries. They suggest that the formation of novel boundaries may involve the accumulation of insulator binding motifs and that lineage-specific boundaries play an important role in TAD reorganization and gene expression, even in the absence of chromosomal rearrangements. The boundaries that we identify as lineage-specific have stronger insulating properties and tend to have more insulator protein binding motifs, compared to their orthologous sequence in the other species (Fig 6B, S5 Table). In addition, the non-boundary orthologs of lineage-specific boundaries show significantly less insulation than the actual boundaries identified in the same species (Fig 6A). Furthermore, our lineage-specific boundaries are reproducible: in order to be lineage-specific, we required boundaries to be independently identified in each replicate for the species of interest and absent from the orthologous sequence in both replicates of the other species. Nevertheless, it remains possible that a portion of the boundaries we identify as lineage-specific are due to inconsistencies in identification between the two species and are actually cases where there is a difference in boundary strength rather than boundary presence/absence. At the very least, we can conservatively conclude that differences in boundary strength (and potentially presence/absence) are associated with differences in the abundance of BEAF-32 and M1BP motifs as well as local changes in gene expression.

Several previous studies have concluded that 3D genome organization is conserved and therefore evolving under purifying selection [2, 7, 23, 25]. These studies have based their conclusion of evolutionary constraint on a statistical correlation of contact frequencies between species, without identifying TADs [7] or on a statistical association between synteny breakpoints and TAD boundaries [2, 23–25]. Renschler et. al. [25] report that 3D genome architecture is conserved across the Drosophila species *D. melanogaster*, *D. virilis*, and *D. busckii* based on a significant association between synteny breakpoints and TAD boundaries, yet find that only 10% of identified TADs were conserved across all three species.

By separating TADs based on chromatin state, we have gained additional insight into their evolution. We find that a subset of TADs are likely evolving under purifying selection, specifically those enriched for developmentally-regulated genes in the BLUE and BLACK chromatin states defined by [53]. Such genes are known to be involved in long-distance physical interactions with enhancers: 4C-seq experiments in Drosophila have shown that developmental enhancers have a high degree of 3D connectivity and form chromatin loops on the order of tens to hundreds of kilobases in size [22]. Reorganization of developmental TAD subtypes should perturb these long-distance contacts and potentially result in the misregulation of developmental genes, which is likely to be deleterious. Accordingly, we find that conserved TADs are enriched for both BLUE and BLACK chromatin states (Fig 7), which are known to mark developmentally-regulated genes, and synteny breakpoints are depleted from these same states (Fig 8A), consistent with selection acting to preserve the organization of these TADs.

On the other hand, we find that TADs enriched for genes with broad expression patterns (i.e. YELLOW chromatin state) are evolving rapidly. Compared to genes in the BLUE and BLACK chromatin states, these genes have a relatively simple regulatory architecture: they are found in genomic regions that are devoid of conserved non-coding elements, and tend to be highly expressed across a variety of tissues [53]. In contrast to the BLUE and BLACK chromatin states, we find that TADs that have been reorganized since the common ancestor of *D. triauraria* and *D. melanogaster* are enriched for genes in the YELLOW chromatin state (Fig 7). We also observe an excess of rearrangement breakpoints in the YELLOW chromatin state for our interspecies comparison versus polymorphic breakpoints from *D. melanogaster* (Fig 8A). These results suggest that natural selection may actually be favoring reorganization of this subtype of TAD. Unlike the BLACK and BLUE chromatin states, we find that reorganized TADs enriched for YELLOW genes contain a significant excess of differentially-expressed genes, raising the possibility that reorganization of these TADs is associated with adaptive changes in gene expression (Fig 8B). Genes previously identified as experiencing adaptive changes in gene expression in Drosophila [58] are highly enriched for the YELLOW chromatin state, suggesting that adaptive evolution of this gene category may be relatively common.

Finally, we also find evidence for a TAD subtype that evolves neutrally. Conserved TADs show a significant depletion of genes from the GREEN chromatin state (Fig 7), which is associated with constitutive heterochromatin, suggesting a lack of evolutionary constraint. However, unlike the YELLOW chromatin state, we find no difference in the abundance of polymorphic versus interspecies rearrangement breakpoints in the GREEN state (Fig 8A), suggesting that rearrangements that disrupt these TADs are accumulating neutrally. Furthermore, reorganization of these TADs is not associated with divergence in gene expression (Fig 8B). Although there are transcribed genes in the GREEN chromatin state, in general it is gene-poor and repeat-rich [53]. The apparently neutral reorganization of these TADs suggests their function could be related more to the packaging of heterochromatin rather than regulation of gene expression.

The evolutionary dynamics of TADs enriched for genes from the RED chromatin state are less clear. This chromatin state is characterized by active euchromatin, but unlike the YELLOW state, genes in the RED state are more likely to have tissue-specific patterns of expression and have a higher density of conserved-noncoding elements [53]. There is a significant depletion of interspecies rearrangements in this chromatin state (Fig 8A), consistent with purifying selection, however, conserved TADs are not enriched for genes from the RED state (Fig 7), possibly because lineage-specific boundaries (which also disrupt TADs) are more likely to form near RED genes. Additionally, in terms of differentially-expressed genes, the RED state is more similar to the YELLOW state, where nonconserved TADs contain a higher fraction of differentially-expressed genes, although the difference is not statistically significant in this case (Fig 8B). It is possible that some TADs containing RED genes are evolving under purifying selection while others are evolving under positive selection.

These results help to reconcile previously contradictory findings regarding TAD evolution. For example, previous comparative studies of TAD organization have either concluded that TADs are highly conserved [2, 7, 23–25] or rapidly evolving (e.g. [26]). Our results show that both conclusions can be true: certain TAD subtypes, such as those containing developmental genes, are likely to be evolving under purifying selection, whereas other TADs, such as those enriched for the YELLOW chromatin state, are evolving rapidly. Additionally, some studies have found that TAD disruption results in aberrant enhancer-promoter contacts and gene misregulation [16, 61] while others have found little to no association between TAD reorganization and differential gene expression [27, 30]. Our results suggest that there are different subtypes of TADs with variable tolerances for disruption. Disruption of some types of TADs, such as those containing developmental genes surrounded by clusters of conserved non-coding regulatory elements (CNEs), may cause widespread alterations of gene expression profiles and these TADs are therefore highly conserved. Other types of TADs, such as those containing fewer CNEs and long-distance enhancer-promoter contacts, could potentially be altered without widespread effects on gene expression. These TADs may be more likely to diverge quickly between species. If this is true, previous studies reporting contradictory effects of TAD rearrangement on gene expression may simply be due to the differences in the subtypes of TAD being tested. Future work involving experimental disruption of an unbiased sample of TADs would allow for testing of this prediction.

## Methods

### *D. triauraria* genome sequencing

Using the Qiagen DNAeasy Blood and Tissue Kit, we extracted DNA from ∼30 *D. triauraria* mated adult females strain 14028-0651.00 (National Drosophila Species Stock Center at Cornell). We used the Oxford Nanopore Technologies (ONT) SQK-LSK 108 library preparation kit to construct three PCR-free libraries according to the ONT 1D Genomic DNA by Ligation protocol. Each library was sequenced on a MinION r9.4 flow cell. Raw signal data were basecalled using the ONT *Albacore* v2.3.4 software package with default parameters.

### Hi-C chromosome conformation capture

*D. triauraria* and *D. melanogaster* strains were maintained in population cages on molasses agar with yeast paste. Embryos (8-16 h) for each species were collected and dechorionated in 50% commercial bleach for 2.5 min. Nuclei were isolated from ∼1 g of embryos and fixed in 1.8% formaldehyde for 15 minutes according to the protocol in Sandmann et. al. [62]. Two replicate Hi-C libraries were constructed for each species using the *in situ* DNase Hi-C protocol described by Ramani et. al. [63]. Libraries were sequenced on an Illumina HiSeq 2500 machine.

### RNA-seq

Approximately 0.02 g fresh embryos were collected using the same approach as for Hi-C libraries and homogenized in 300 *μL* 1x DNA/RNA Shield. Fifty pairs of testes were dissected from 3-5-day old mated males and 10 pairs of ovaries from 3-5-day old mated females. Dissections were performed in 1X PBS and then immediately transferred into 200 *μL* RNAlater solution. Two hundred *μL* of ice-cold 1X PBS was added to each sample and they were centrifuged at 5000g for 1 min at 4°C. After removing the supernatant, 300 *μL* 1x DNA/RNA shield was added and samples were homogenized immediately using an electric pestle.

RNA was extracted using the Quick-RNA Plus Kit (R1057) from Zymo Research. Samples were incubated at 55°C with 30 *μL* PK Digestion Buffer and 15 *μL* Proteinase K for at least 30 minutes. Column-based size selection was used to obtain >200 nt purified total RNA. MRNA-seq libraries were constructed from the total RNA using the NEBNext Poly(A) mRNA Magnetic Isolation Module (E7049) and the NEBNext Ultra II Directional RNA Library Prep Kit for Illumina (E7760) using 1 *μg* total RNA with fragmentation for 7 min at 94°C and first strand cDNA synthesis via incubation for 45 minutes at 42°C. Library quality was assessed on a Bioanalyzer. For the embryo samples, two biological replicate libraries were prepared for *D. triauraria* strain 14028-0651.00 (National Drosophila Species Stock Center at Cornell) while *D. melanogaster* strains RAL-379 and RAL-732 from the Drosophila Genetic Reference Panel (DGRP) [64] were used as biological replicates for *D. melanogaster*.

### *D. triauraria* genome assembly and annotation

The basecalled *D. triauraria* nanopore reads were combined with the nanopore reads from Miller et. al. [34] and assembled using *Canu* [35]. *Purge Haplotigs* [38] was used to identify and collapse heterozygous contigs where each haplotype was assembled separately. The assembly was then polished using the raw nanopore signal data with *Nanopolish* [36]. Finally, uninformative Hi-C Illumina sequences (i.e. those that do not contain a ligation junction) were used as single-end reads to further polish the assembly with *Pilon* [37]. To confirm that our data were from the same *D. triauraria* strain as the data generated by Miller et. al. [34], we aligned our uninformative *D. triauraria* Hi-C reads and the *D. triauraria* Illumina data from Miller et. al. (SRA project PRJNA473618) [34] as single-end reads using *bowtie2* (version 2.2.9) [65] with default parameters. We then called SNPs using *Freebayes* (version 1.2.0) [44] with the parameters *–no-indels –no-mnps –no-complex -0 –report-monomorphic –use-best-n-alleles 4*.

The *Juicer* [39] and *3D-DNA* [40] pipelines were used to scaffold the *D. triauraria* nanopore reads with Hi-C sequencing data. The *Juicebox* software package [66] was used to visualize contact matrices, assign chromosome boundaries, and export a finalized reference sequence for downstream analysis.

To assign the chromosome-length *D. triauraria* scaffolds to their corresponding Muller element (i.e. Muller A-F), we performed a translated BLAST search of our scaffolds using FlyBase r6.21 [67] *D. melanogaster* peptides as queries (Fig 2A and 2B).

To pre-process the RNA-seq data for *MAKER* [43], we first aligned the *D. triauraria* RNA-seq Illumina reads to the newly assembled *D. triauraria* reference genome using *HISAT2* [68]. Second, the *HISAT2* alignments were used to assemble mRNA transcripts with *stringtie* [69]. The *stringtie* transcripts were provided to *MAKER* along with *D. melanogaster* r6.26 peptides from FlyBase [67]. The *MAKER* control file is available via GitHub.

### Genome synteny

We identified synteny blocks between *D. melanogaster* and *D. triauraria* using *Mercator* [46] software. We visualized synteny using the promer tools from the *MUMmer* [47] pipeline to produce a dotplot comparison of the *D. melanogaster* and *D. triauraria* genomes.

### Identifying TAD boundaries and domains

We removed adapter sequences from Hi-C reads for each species using *Trimmomatic* [70] and used a custom perl script to split reads that contain a ligation junction. We used *BWA* software [71] to align the split forward and reverse Hi-C reads to each species’ reference assembly (the *D.triauraria* assembly generated in this study and the *D. melanogaster* release 6 assembly from Flybase [67]). We used *HiCExplorer* version 2.2 [10] to create a normalized contact frequency matrix. To find TAD boundaries and domains for each species we ran the *hicFindTads* utility separately for each biological replicate. We used *Bedtools* [72] to identify overlapping boundaries and domains between replicates. Boundaries were required to overlap by at least one base pair in both replicates. Domains were required to have start and end coordinates within 5000 bp in both replicates. Boundaries and domains identified in both replicates were considered high confidence while those identified in one replicate are low confidence. We used a custom python script to calculate correlation coefficients between replicates for the TAD separation scores. Boundary and intra-TAD insulation scores were calculated for 5 kb bins using *HiCExplorer*.

### Defining and identifying orthologous TAD boundaries between *D. melanogaster* and *D. triauraria*

We softmasked the *D. melanogaster* and *D. triauraria* genomes using *Repeatmasker* [73] and aligned them using *Cactus* [50] to generate a hal file. We input the high confidence boundaries for *D. melanogaster* to *halLiftover* [51] to identify the corresponding genomic coordinates in *D. triauraria*. *HalLiftover* reports contiguous liftover coordinates as separate features if they include short indels. We therefore merged ‘lifted-over’ boundary locations that were within 5000 bp of each other into a single feature. Lifted-over boundaries less than 500 bp in size were excluded from further analysis. We considered lifted-over boundaries from *D. triauraria* that were located less than 5 kb from either a high or low confidence boundary in *D. melanogaster* to be orthologous boundaries. Lifted-over boundaries from *D. triauraria* that were not identified as boundaries in *D. melanogaster* were considered non-orthologous. We implemented the same pipeline for the reverse comparison, from *D. triauraria* to *D. melanogaster*. We defined a lineage-specific boundary as a high-confidence boundary present in one species whose orthologous sequence in the other species did not overlap either a high- or low-confidence boundary.

### Boundary motif enrichment

We used *Homer* [49] software to search for enriched motifs with the high-confidence boundary sequences for both *D. triauraria* and *D. melanogaster*. We split each genome assembly into 5 kb sequences for use as the background dataset. To compare motif occurrences between lineage-specific boundaries and their orthologs, we downloaded motif matrix profiles from JASPAR [74] for BEAF-32 (accession MA0529.2) and M1BP (accession MA1459.1) and used FIMO [75] to identify occurrences of each motif within lineage-specific boundaries as well as their orthologs. We summed the number of nonoverlapping motif occurrences within each boundary and compared the number of motifs in each lineage-specific boundary to the number of occurrences of the motif in the orthologous non-boundary sequence.

### Defining and identifying orthologous domains between *D. melanogaster* and *D. triauraria*

To assess domain conservation between *D. melanogaster* and *D. triauraria* we used *halLiftover* [51]. *HalLiftover* will report lifted-over coordinates as separate features if there are species-specific indels, transposon insertions, or chromosomal rearrangements. In order to combine contiguously lifted-over segments that were separated by species-specific indels, TE insertions, or intra-TAD rearrangements, we merged lifted-over features separated by less than 20 kb. Lifted-over features less than 5000 bp were excluded from further analysis. After merging, the lifted-over domains in *D. triauraria* that reciprocally overlapped a *D. melanogaster* high or low confidence domain (>90%) were considered orthologous domains.

Lifted-over domains in *D. triauraria* that did not meet the reciprocal overlap criteria with a *D. melanogaster* domain were considered non-orthologous. To identify orthologous domains between the two species that also share boundaries, we required the *D. triauraria* lifted-over endpoints to lie within 5 kb of the *D. melanogaster* orthologous TAD boundaries. Non-orthologous domains were categorized into truncated/expanded, or split domains. Truncated and expanded domains are not split by chromosomal rearrangements or lineage-specific boundaries, rather, they do not meet the 90% reciprocal overlap criterion due to large insertion/deletion mutations that have created asymmetry in TAD size between the two species. Split domains included those split by rearrangement and lineage-specific boundaries. We implemented the same pipeline for the reverse comparison, from *D. triauraria* to *D. melanogaster*. To determine the number of co-linear TADs and boundaries expected by chance, we used *Bedtools* to shuffle their locations and determine how many of the shuffled features were located entirely within a synteny block. We then calculated the average number of co-linear TADs/boundaries across 100 shuffles.

### Gene expression

Stranded embryo RNA-seq data were aligned to their respective genomes using *HISAT2* (version 2.1.0) [68] with parameters *–dta –max-intronlen 50000 –rna-strandness RF*. Per-gene raw read counts were generated using *htseq-count* (version 0.11.2) [76] with parameters *-i Parent -f bam -r pos -s reverse -a 20 –nonunique none*. Our *MAKER* [43] gene models were used for *D. triauraria* and the FlyBase r6.21 [67] gene models were used for *D. melanogaster*. One-to-one gene orthologs were identified using our *Mercator* [46] orthology map and differentially-expressed genes were identified using the *DESeq2* R software package [52].

### Chromatin state

We used the chromatin state annotations from Filion et. al. [53] to assign each *D. melanogaster* gene to one of five chromatin states (BLACK, BLUE, GREEN, RED, YELLOW). Genes were assigned to chromatin states based on the state that covered the largest proportion of the gene (including introns) and we counted the number of genes from orthologous versus non-orthologous TADs for each of the five chromatin states. To determine whether genes from the RED chromatin state tend to be near lineage-specific boundaries, we used *Bedtools* to determine the number of genes from the RED chromatin state that overlapped a lineage-specific boundary. We then used *Bedtools* to shuffle the lineage-specific boundary locations and counted the number of genes from the RED chromatin state that overlapped a shuffled boundary. We performed 1000 shuffles in total.

### Rearrangement breakpoints

We used Mercator to identify synteny breakpoints between *D. melanogaster* and *D. triauraria*. To compare interspecies breakpoint locations to intraspecies rearrangements, we used the locations of polymorphic *D. melanogaster* inversions that were 10 kb or larger, identified from long-read sequencing of 14 *D. melanogaster strains* [57].

### *D. triauraria* polymorphic inversion breakpoints

We visually identified polymorphic inversion breakpoints using the *Juicebox* software package and estimated coordinate. Polymorphic inversions are evident through “bow-tie” like contact points accompanied by high contact frequencies along the diagonal at the breakpoint. To confirm that polymorphic inversion breakpoints did not disrupt TAD boundaries we intersected the coordinates with high confidence *D. triauraria* boundaries and calculated the number of breakpoints that intersected boundaries and the median distance of breakpoints from boundaries. Additionally, we ran 1000 permutations of shuffled inversion breakpoints to compare expected number of breakpoint/boundary intersections and median distance from TAD boundary to the observed values.

## Supporting information

S1 Fig Muller element genes per *D. triauraria* megascaffold. Percent of *D. melanogaster* genes corresponding to each of the *D. triauraria* chromosome-length scaffolds. Each scaffold is enriched for genes belonging to a single Muller element.
(TIF)

S2 Fig *D. triauraria* polymorphic inversions. A) Muller B B) Muller C and C) Muller D. Light blue dots represent inversion breakpoints, black lines outline entire inverted sections. Dark blue rectangles highlight high contact frequencies along the diagonal demonstrating that inversions are polymorphic.
(TIF)

S3 Fig Distribution of the number of nanopore reads spanning *D. triauraria* inversion breakpoints. (TIF)

S4 Fig Scatterplots depicting the correlation between TAD separation score between replicate datasets. TAD separation scores from each replicate plotted for A) *D. melanogaster* (Spearman’s rho: 0.995) and B) *D. triauraria* (Spearman’s rho: 0.990).
(TIF)

S5 Fig Sequence logo diagrams for enriched motifs at TAD boundaries. Motifs for A) *D. melanogaster* M1BP (*p* = 1e–17), B) *D. melanogaster* BEAF-32/DREF (*p* = 1e-18), C) *D. triauraria* M1BP (*p* = 1e–42), and D) *D. triauraria* BEAF-32/DREF (*p* = 1e–15). *Homer* [49] software found these sequence motifs to be enriched at TAD boundaries in *D. melanogster* and *D. triauraria*. BEAF-32 and DREF binding motifs are almost identical and both are recognized by the BEAF-32 insulator protein [77].
(TIF)

S6 Fig The X chromosome has more chromosomal rearrangements and fewer orthologous TADs, compared to autosomes. (A) The size of *D. melanogaster* synteny blocks is significantly reduced on the X chromosome (Muller A) relative to the autosomes, indicating that the X has accumulated more chromosomal rearrangements (Wilcoxon test *p* = 6.7e–05). (B) The proportion of orthologous lifted-over TAD domains is also significantly reduced on the X chromosome (Fisher’s Exact Test *p* = 0.0135).
(TIF)

S7 Fig MA plot highlighting differentially-expressed genes between *D. triauraria* and *D. melanogaster*. Differentially-expressed genes (adjusted p-value <= 0.05) between *D. melanogaster* and *D. triauraria* identified by *DEseq2* [52] are shown in red. Each point represents an orthologous gene pair between the two species. The plot was created using the *DEseq2* shrunken log2 fold changes which removes noise from low count genes.
(TIF)

S8 Fig Genes in RED chromatin state overlap lineage-specific boundaries more often than expected by chance. Permutation *p* < 0.001.
(TIF)

S9 Fig Plot with full data range for Fig 6B. Outlier points were removed from Fig 6B to aid in visualization. All datapoints are shown here.
(TIF)

S1 Table Combined *D. triauraria* sequencing and assembly data. This study and Miller et al. [34]. The decrease in assembly size after scaffolding is mainly due to the removal of contigs by *Purge haplotigs* [38].
(PDF)

S2 Table Total number of Hi-C read pairs for each species and replicate, the number of pairs considered and used by *HiCExplorer* [10]. (PDF)

S3 Table Counts of high and low confidence TAD boundaries and domains identified in *D. melanogaster* and *D. triauraria*. (PDF)

S4 Table Summary of results from *D. melanogaster* to *D. triauraria* liftover analysis. (PDF)

S5 Table Lineage-specific boundaries have significantly more insulator protein (BEAF-32 and M1BP) binding motifs compared to the orthologous region in the other species. Paired Wilcoxon test *p* = 0.0059.
(PDF)

S6 Table Number of genes of each chromatin state in orthologous and non-orthologous TADs. (PDF)

## References

[1] Lieberman-Aiden Erez, van Berkum Nynke L, Williams Louise, Imakaev Maxim, Ragoczy Tobias, Telling Agnes, Amit Ido, Lajoie Bryan R, Sabo Peter J, Dorschner Michael O, Sandstrom Richard, Bernstein Bradley, Bender M A, Groudine Mark, Gnirke Andreas, Stamatoyannopoulos John, Mirny Leonid A, Lander Eric S, and Dekker Job. Comprehensive mapping of long-range interactions reveals folding principles of the human genome. *Science*, 326(5950):289–293, 10 2009 10.1126/science.1181369 19815776 PMC2858594

[2] Dixon Jesse R, Selvaraj Siddarth, Yue Feng, Kim Audrey, Li Yan, Shen Yin, Hu Ming, Liu Jun S, and Ren Bing. Topological domains in mammalian genomes identified by analysis of chromatin interactions. *Nature*, 485(7398):376–380, 4 2012 10.1038/nature11082 22495300 PMC3356448

[3] Sexton Tom, Yaffe Eitan, Kenigsberg Ephraim, Bantignies Frédéric, Leblanc Benjamin, Hoichman Michael, Parrinello Hugues, Tanay Amos, and Cavalli Giacomo. Three-dimensional folding and functional organization principles of the *Drosophila* genome. *Cell*, 148(3):458–472, 2 2012 10.1016/j.cell.2012.01.010 22265598

[4] Schauer Tamás, Ghavi-Helm Yad, Sexton Tom, Albig Christian, Regnard Catherine, Cavalli Giacomo, Furlong Eileen Em, and Becker Peter B. Chromosome topology guides the drosophila dosage compensation complex for target gene activation. *EMBO Rep.*, 8 2017 10.15252/embr.201744292 28794204 PMC5623837

[5] Stadler Michael R, Haines Jenna E, and Eisen Michael B. Convergence of topological domain boundaries, insulators, and polytene interbands revealed by high-resolution mapping of chromatin contacts in the early *Drosophila melanogaster* embryo. *Elife*, 6, 11 2017 10.7554/eLife.29550 29148971 PMC5739541

[6] Rao Suhas S P, Huang Su-Chen, Hilaire Brian Glenn St, Engreitz Jesse M, Perez Elizabeth M, Kieffer-Kwon Kyong-Rim, Sanborn Adrian L, Johnstone Sarah E, Bascom Gavin D, Bochkov Ivan D, Huang Xingfan, Shamim Muhammad S, Shin Jaeweon, Turner Douglass, Ye Ziyi, Omer Arina D, Robinson James T, Schlick Tamar, Bernstein Bradley E, Casellas Rafael, Lander Eric S, and Aiden Erez Lieberman. Cohesin loss eliminates all loop domains. *Cell*, 171(2):305–320.e24, 10 2017 10.1016/j.cell.2017.09.026 28985562 PMC5846482

[7] Rudan Matteo Vietri, Barrington Christopher, Henderson Stephen, Ernst Christina, Odom Duncan T, Tanay Amos, and Hadjur Suzana. Comparative Hi-C reveals that CTCF underlies evolution of chromosomal domain architecture. *Cell Rep.*, 10(8):1297–1309, 3 2015 10.1016/j.celrep.2015.02.004 25732821 PMC4542312

[8] Phillips-Cremins Jennifer E, Sauria Michael E G, Sanyal Amartya, Gerasimova Tatiana I, Lajoie Bryan R, Bell Joshua S K, Ong Chin-Tong, Hookway Tracy A, Guo Changying, Sun Yuhua, Bland Michael J, Wagstaff William, Dalton Stephen, McDevitt Todd C, Sen Ranjan, Dekker Job, Taylor James, and Corces Victor G. Architectural protein subclasses shape 3D organization of genomes during lineage commitment. *Cell*, 153(6):1281–1295, 6 2013 10.1016/j.cell.2013.04.053 23706625 PMC3712340

[9] Szabo Quentin, Bantignies Frédéric, and Cavalli Giacomo. Principles of genome folding into topologically associating domains. *Sci Adv*, 5(4):eaaw1668, 4 2019 10.1126/sciadv.aaw1668 30989119 PMC6457944

[10] Ramírez Fidel, Bhardwaj Vivek, Arrigoni Laura, Lam Kin Chung, Grüning Björn A, Villaveces José, Habermann Bianca, Akhtar Asifa, and Manke Thomas. High-resolution TADs reveal DNA sequences underlying genome organization in flies. *Nat. Commun.*, 9(1):189, 1 2018 10.1038/s41467-017-02525-w 29335486 PMC5768762

[11] Hug Clemens B, Grimaldi Alexis G, Kruse Kai, and Vaquerizas Juan M. Chromatin architecture emerges during zygotic genome activation independent of transcription. *Cell*, 169(2):216–228.e19, 4 2017 10.1016/j.cell.2017.03.024 28388407

[12] Hou Chunhui, Li Li, Qin Zhaohui S, and Corces Victor G. Gene density, transcription, and insulators contribute to the partition of the drosophila genome into physical domains. *Mol. Cell*, 48(3):471–484, 11 2012 10.1016/j.molcel.2012.08.031 23041285 PMC3496039

[13] Ulianov Sergey V, Khrameeva Ekaterina E, Gavrilov Alexey A, Flyamer Ilya M, Kos Pavel, Mikhaleva Elena A, Penin Aleksey A, Logacheva Maria D, Imakaev Maxim V, Chertovich Alexander, Gelfand Mikhail S, Shevelyov Yuri Y, and Razin Sergey V. Active chromatin and transcription play a key role in chromosome partitioning into topologically associating domains. *Genome Res.*, 26(1):70–84, 1 2016 10.1101/gr.196006.115 26518482 PMC4691752

[14] Bonev Boyan, Cohen Netta Mendelson, Szabo Quentin, Fritsch Lauriane, Papadopoulos Giorgio L, Lubling Yaniv, Xu Xiaole, Lv Xiaodan, Hugnot Jean-Philippe, Tanay Amos, and Cavalli Giacomo. Multiscale 3D genome rewiring during mouse neural development. *Cell*, 171(3):557–572.e24, 10 2017 10.1016/j.cell.2017.09.043 29053968 PMC5651218

[15] Symmons Orsolya, Uslu Veli Vural, Tsujimura Taro, Ruf Sandra, Nassari Sonya, Schwarzer Wibke, Ettwiller Laurence, and Spitz François. Functional and topological characteristics of mammalian regulatory domains. *Genome Res.*, 24(3):390–400, 3 2014 10.1101/gr.163519.113 24398455 PMC3941104

[16] Lupiáñez Darío G, Kraft Katerina, Heinrich Verena, Krawitz Peter, Brancati Francesco, Klopocki Eva, Horn Denise, Kayserili Hülya, Opitz John M, Laxova Renata, Santos-Simarro Fernando, Gilbert-Dussardier Brigitte, Wittler Lars, Borschiwer Marina, Haas Stefan A, Osterwalder Marco, Franke Martin, Timmermann Bernd, Hecht Jochen, Spielmann Malte, Visel Axel, and Mundlos Stefan. Disruptions of topological chromatin domains cause pathogenic rewiring of gene-enhancer interactions. *Cell*, 161(5):1012–1025, 5 2015 10.1016/j.cell.2015.04.004 25959774 PMC4791538

[17] Franke Martin, Ibrahim Daniel M, Andrey Guillaume, Schwarzer Wibke, Heinrich Verena, Schöpflin Robert, Kraft Katerina, Kempfer Rieke, Jerković Ivana, Chan Wing-Lee, Spielmann Malte, Timmermann Bernd, Wittler Lars, Kurth Ingo, Cambiaso Paola, Zuffardi Orsetta, Houge Gunnar, Lambie Lindsay, Brancati Francesco, Pombo Ana, Vingron Martin, Spitz Francois, and Mundlos Stefan. Formation of new chromatin domains determines pathogenicity of genomic duplications. *Nature*, 538(7624):265–269, 10 2016 10.1038/nature19800 27706140

[18] Flavahan William A, Drier Yotam, Liau Brian B, Gillespie Shawn M, Venteicher Andrew S, Stemmer-Rachamimov Anat O, Suvà Mario L, and Bernstein Bradley E. Insulator dysfunction and oncogene activation in IDH mutant gliomas. *Nature*, 529(7584):110–114, 1 2016 10.1038/nature16490 26700815 PMC4831574

[19] Hnisz Denes, Weintraub Abraham S, Day Daniel S, Valton Anne-Laure, Bak Rasmus O, Li Charles H, Goldmann Johanna, Lajoie Bryan R, Fan Zi Peng, Sigova Alla A, Reddy Jessica, Borges-Rivera Diego, Lee Tong Ihn, Jaenisch Rudolf, Porteus Matthew H, Dekker Job, and Young Richard A. Activation of proto-oncogenes by disruption of chromosome neighborhoods. *Science*, 351(6280):1454–1458, 3 2016 10.1126/science.aad9024 26940867 PMC4884612

[20] Lupiáñez Darío G, Spielmann Malte, and Mundlos Stefan. Breaking TADs: How alterations of chromatin domains result in disease. *Trends Genet.*, 32(4):225–237, 4 2016 10.1016/j.tig.2016.01.003 26862051

[21] Weischenfeldt Joachim, Dubash Taronish, Drainas Alexandros P, Mardin Balca R, Chen Yuanyuan, Stütz Adrian M, Waszak Sebastian M, Bosco Graziella, Halvorsen Ann Rita, Raeder Benjamin, Efthymiopoulos Theocharis, Erkek Serap, Siegl Christine, Brenner Hermann, Brustugun Odd Terje, Dieter Sebastian M, Northcott Paul A, Petersen Iver, Pfister Stefan M, Schneider Martin, Solberg Steinar K, Thunissen Erik, Weichert Wilko, Zichner Thomas, Thomas Roman, Peifer Martin, Helland Aslaug, Ball Claudia R, Jechlinger Martin, Sotillo Rocio, Glimm Hanno, and Korbel Jan O. Pan-cancer analysis of somatic copy-number alterations implicates IRS4 and IGF2 in enhancer hijacking. *Nat. Genet.*, 49(1):65–74, 1 2017 10.1038/ng.3722 27869826 PMC5791882

[22] Ghavi-Helm Yad, Klein Felix A, Pakozdi Tibor, Ciglar Lucia, Noordermeer Daan, Huber Wolfgang, and Furlong Eileen E M. Enhancer loops appear stable during development and are associated with paused polymerase. *Nature*, 512(7512):96–100, 8 2014 10.1038/nature13417 25043061

[23] Krefting Jan, Andrade-Navarro Miguel A, and Ibn-Salem Jonas. Evolutionary stability of topologically associating domains is associated with conserved gene regulation. *BMC Biol.*, 16(1):87, 8 2018 10.1186/s12915-018-0556-x 30086749 PMC6091198

[24] Lazar Nathan H, Nevonen Kimberly A, O’Connell Brendan, McCann Christine, O’Neill Rachel J, Green Richard E, Meyer Thomas J, Okhovat Mariam, and Carbone Lucia. Epigenetic maintenance of topological domains in the highly rearranged gibbon genome. *Genome Res.*, 28(7):983–997, 7 2018 10.1101/gr.233874.117 29914971 PMC6028127

[25] Renschler Gina, Richard Gautier, Valsecchi Claudia Isabelle Keller, Toscano Sarah, Arrigoni Laura, Ramirez Fidel, and Akhtar Asifa. Hi -C guided assemblies reveal conserved regulatory topologies on X and autosomes despite extensive genome shuffling. *BioRxiv*, 3 2019. 10.1101/gad.328971.119 PMC6824461 31601616

[26] Eres Ittai E, Luo Kaixuan, Hsiao Chiaowen Joyce, Blake Lauren E, and Gilad Yoav. Reorganization of 3D genome structure may contribute to gene regulatory evolution in primates. *PLoS Genet.*, 15(7):e1008278, 7 2019 10.1371/journal.pgen.1008278 31323043 PMC6668850

[27] Ghavi-Helm Yad, Jankowski Aleksander, Meiers Sascha, Viales Rebecca R, Korbel Jan O, and Furlong Eileen E M. Highly rearranged chromosomes reveal uncoupling between genome topology and gene expression. *Nat. Genet.*, 51(8):1272–1282, 8 2019 10.1038/s41588-019-0462-3 31308546 PMC7116017

[28] Lee Hangnoh, Cho Dong-Yeon, Whitworth Cale, Eisman Robert, Phelps Melissa, Roote John, Kaufman Thomas, Cook Kevin, Russell Steven, Przytycka Teresa, and Oliver Brian. Effects of gene dose, chromatin, and network topology on expression in *Drosophila melanogaster*. *PLoS Genet.*, 12(9):e1006295, 9 2016 10.1371/journal.pgen.1006295 27599372 PMC5012587

[29] Meadows Lisa A, Chan Yuk Sang, Roote John, and Russell Steven. Neighbourhood continuity is not required for correct testis gene expression in *Drosophila*. *PLoS Biol.*, 8(11):e1000552, 11 2010 10.1371/journal.pbio.1000552 21151342 PMC2994658

[30] Despang Alexandra, Schöpflin Robert, Franke Martin, Ali Salaheddine, Jerković Ivana, Paliou Christina, Chan Wing-Lee, Timmermann Bernd, Wittler Lars, Vingron Martin, Mundlos Stefan, and Ibrahim Daniel M. Functional dissection of the Sox9-Kcnj2 locus identifies nonessential and instructive roles of TAD architecture. *Nat. Genet.*, 51(8):1263–1271, 8 2019 10.1038/s41588-019-0466-z 31358994

[31] Harmston Nathan, Ing-Simmons Elizabeth, Tan Ge, Perry Malcolm, Merkenschlager Matthias, and Lenhard Boris. Topologically associating domains are ancient features that coincide with metazoan clusters of extreme noncoding conservation. *Nat. Commun.*, 8(1):441, 9 2017 10.1038/s41467-017-00524-5 28874668 PMC5585340

[32] Obbard Darren J, Maclennan John, Kim Kang-Wook, Rambaut Andrew, O’Grady Patrick M, and Jiggins Francis M. Estimating divergence dates and substitution rates in the drosophila phylogeny. *Mol. Biol. Evol.*, 29(11):3459–3473, 11 2012 10.1093/molbev/mss150 22683811 PMC3472498

[33] Bhutkar Arjun, Schaeffer Stephen W, Russo Susan M, Xu Mu, Smith Temple F, and Gelbart William M. Chromosomal rearrangement inferred from comparisons of 12 drosophila genomes. *Genetics*, 179(3):1657–1680, 7 2008 10.1534/genetics.107.086108 18622036 PMC2475759

[34] Miller Danny E, Staber Cynthia, Zeitlinger Julia, and Hawley R Scott. Highly contiguous genome assemblies of 15 *drosophila* species generated using nanopore sequencing. *G3*, 8(10):3131–3141, 10 2018 10.1534/g3.118.200160 30087105 PMC6169393

[35] Koren Sergey, Walenz Brian P, Berlin Konstantin, Miller Jason R, Bergman Nicholas H, and Phillippy Adam M. Canu: scalable and accurate long-read assembly via adaptive k-mer weighting and repeat separation. *Genome Res.*, 27(5):722–736, 5 2017 10.1101/gr.215087.116 28298431 PMC5411767

[36] Loman Nicholas J, Quick Joshua, and Simpson Jared T. A complete bacterial genome assembled de novo using only nanopore sequencing data. *Nat. Methods*, 12(8):733–735, 8 2015 10.1038/nmeth.3444 26076426

[37] Walker Bruce J, Abeel Thomas, Shea Terrance, Priest Margaret, Abouelliel Amr, Sakthikumar Sharadha, Cuomo Christina A, Zeng Qiandong, Wortman Jennifer, Young Sarah K, and Earl Ashlee M. Pilon: an integrated tool for comprehensive microbial variant detection and genome assembly improvement. *PLoS One*, 9(11):e112963, 11 2014 10.1371/journal.pone.0112963 25409509 PMC4237348

[38] Roach Michael J, Schmidt Simon A, and Borneman Anthony R. Purge haplotigs: allelic contig reassignment for third-gen diploid genome assemblies. *BMC Bioinformatics*, 19(1):460, 11 2018 10.1186/s12859-018-2485-7 30497373 PMC6267036

[39] Durand Neva C, Shamim Muhammad S, Machol Ida, Rao Suhas S P, Huntley Miriam H, Lander Eric S, and Aiden Erez Lieberman. Juicer provides a one-click system for analyzing loop-resolution Hi-C experiments. *Cell systems*, 3:95–98, 7 2016 10.1016/j.cels.2016.07.002 27467249 PMC5846465

[40] Dudchenko Olga, Batra Sanjit S, Omer Arina D, Nyquist Sarah K, Hoeger Marie, Durand Neva C, Shamim Muhammad S, Machol Ido, Lander Eric S, Aiden Aviva Presser, and Aiden Erez Lieberman. De novo assembly of the *Aedes aegypti* genome using Hi-C yields chromosome-length scaffolds. *Science*, 356(6333):92–95, 4 2017 10.1126/science.aal3327 28336562 PMC5635820

[41] Stanke Mario and Waack Stephan. Gene prediction with a hidden markov model and a new intron submodel. *Bioinformatics*, 19 Suppl 2:ii215–25, 10 2003 14534192 10.1093/bioinformatics/btg1080

[42] Korf Ian. Gene finding in novel genomes. *BMC Bioinformatics*, 5:59, 5 2004 10.1186/1471-2105-5-59 15144565 PMC421630

[43] Cantarel Brandi L, Korf Ian, Robb Sofia M C, Parra Genis, Ross Eric, Moore Barry, Holt Carson, Alvarado Alejandro Sánchez, and Yandell Mark. MAKER: an easy-to-use annotation pipeline designed for emerging model organism genomes. *Genome Res.*, 18(1):188–196, 1 2008 10.1101/gr.6743907 18025269 PMC2134774

[44] Garrison Erik and Marth Gabor. Haplotype-based variant detection from short-read sequencing. *arXiv [q-bio.GN]*, pages 1–9, 7 2012.

[45] Mavragani-Tsipidou P, Scouras G, Haralampidis K, Lavrentiadou S, and Kastritsis C D. The polytene chromosomes of *Drosophila triauraria* and *D. quadraria*, sibling species of *D. auraria*. *Genome*, 1984:318–327, 1992. 10.1139/g92-048 1618391

[46] Dewey Colin N. Aligning multiple whole genomes with mercator and MAVID. *Methods Mol. Biol.*, 395:221–236, 2007 10.1007/978-1-59745-514-5_14 17993677

[47] Kurtz Stefan, Phillippy Adam, Delcher Arthur L, Smoot Michael, Shumway Martin, Antonescu Corina, and Salzberg Steven L. Versatile and open software for comparing large genomes. *Genome Biol.*, 5(2):R12, 1 2004 10.1186/gb-2004-5-2-r12 14759262 PMC395750

[48] Schaeffer Stephen W, Bhutkar Arjun, McAllister Bryant F, Matsuda Muneo, Matzkin Luciano M, O’Grady Patrick M, Rohde Claudia, Valente Vera L S, Aguadé Montserrat, Anderson Wyatt W, Edwards Kevin, Garcia Ana C L, Goodman Josh, Hartigan James, Kataoka Eiko, Lapoint Richard T, Lozovsky Elena R, Machado Carlos A, Noor Mohamed A F, Papaceit Montserrat, Reed Laura K, Richards Stephen, Rieger Tania T, Russo Susan M, Sato Hajime, Segarra Carmen, Smith Douglas R, Smith Temple F, Strelets Victor, Tobari Yoshiko N, Tomimura Yoshihiko, Wasserman Marvin, Watts Thomas, Wilson Robert, Yoshida Kiyohito, Markow Therese A, Gelbart William M, and Kaufman Thomas C. Polytene chromosomal maps of 11 Drosophila species: the order of genomic scaffolds inferred from genetic and physical maps. *Genetics*, 179(3):1601–1655, 7 2008 10.1534/genetics.107.086074 18622037 PMC2475758

[49] Heinz Sven, Benner Christopher, Spann Nathanael, Bertolino Eric, Lin Yin C, Laslo Peter, Cheng Jason X, Murre Cornelis, Singh Harinder, and Glass Christopher K. Simple combinations of lineage-determining transcription factors prime cis-regulatory elements required for macrophage and B cell identities. *Mol. Cell*, 38(4):576–589, 5 2010 10.1016/j.molcel.2010.05.004 20513432 PMC2898526

[50] Joel Armstrong, Glenn Hickey, Mark Diekhans, Alden Deran, Qi Fang, Duo Xie, Shaohong Feng, Josefin Stiller, Diane Genereux, Jeremy Johnson, Voichita Dana Marinescu, David Haussler, Jessica Alföldi, Kerstin Lindblad-Toh, Elinor Karlsson, Guojie Zhang, and Benedict Paten. Progressive alignment with Cactus: a multiple-genome aligner for the thousand-genome era. August 2019.

[51] Hickey Glenn, Paten Benedict, Earl Dent, Zerbino Daniel, and Haussler David. HAL: a hierarchical format for storing and analyzing multiple genome alignments. *Bioinformatics*, 29(10):1341–1342, 5 2013 10.1093/bioinformatics/btt128 23505295 PMC3654707

[52] Anders Simon and Huber Wolfgang. Differential expression analysis for sequence count data. *Genome Biol.*, 11(10):R106, 10 2010 10.1186/gb-2010-11-10-r106 20979621 PMC3218662

[53] Filion Guillaume J, Bemmel Joke G van, Braunschweig Ulrich, Talhout Wendy, Kind Jop, Ward Lucas D, Brugman Wim, Castro Inês J de, Kerkhoven Ron M, Bussemaker Harmen J, and Steensel Bas van. Systematic protein location mapping reveals five principal chromatin types in drosophila cells. *Cell*, 143(2):212–224, 10 2010 10.1016/j.cell.2010.09.009 20888037 PMC3119929

[54] Lyne Rachel, Smith Richard, Rutherford Kim, Wakeling Matthew, Varley Andrew, Guillier Francois, Janssens Hilde, Ji Wenyan, Mclaren Peter, North Philip, Rana Debashis, Riley Tom, Sullivan Julie, Watkins Xavier, Woodbridge Mark, Lilley Kathryn, Russell Steve, Ashburner Michael, Mizuguchi Kenji, and Micklem Gos. FlyMine: an integrated database for drosophila and anopheles genomics. *Genome Biol.*, 8(7):R129, 2007 10.1186/gb-2007-8-7-r129 17615057 PMC2323218

[55] Bredesen Bjørn André and Rehmsmeier Marc. DNA sequence models of genome-wide Drosophila melanogaster Polycomb binding sites improve generalization to independent Polycomb Response Elements. *Nucleic Acids Res.*, 47(15):7781–7797, 9 2019 10.1093/nar/gkz617 31340029 PMC6735708

[56] Kimura M. Evolutionary rate at the molecular level. *Nature*, 217(5129):624–626, 2 1968 10.1038/217624a0 5637732

[57] Chakraborty Mahul, Emerson J J, Macdonald Stuart J, and Long Anthony D. Structural variants exhibit widespread allelic heterogeneity and shape variation in complex traits. *Nat. Commun.*, 10(1):4872, 10 2019 10.1038/s41467-019-12884-1 31653862 PMC6814777

[58] Nourmohammad Armita, Rambeau Joachim, Held Torsten, Kovacova Viera, Berg Johannes, and Lässig Michael. Adaptive evolution of gene expression in drosophila. *Cell Rep.*, 20(6):1385–1395, 8 2017 10.1016/j.celrep.2017.07.033 28793262

[59] Ellison Christopher E and Cao Weihuan. Nanopore sequencing and Hi-C scaffolding provide insight into the evolutionary dynamics of transposable elements and piRNA production in wild strains of drosophila melanogaster. *Nucleic Acids Res.*, 48(1):290–303, 1 2020 10.1093/nar/gkz1080 31754714 PMC6943127

[60] Zufferey Marie, Tavernari Daniele, Oricchio Elisa, and Ciriello Giovanni. Comparison of computational methods for the identification of topologically associating domains. *Genome Biol.*, 19(1):217, 12 2018 10.1186/s13059-018-1596-9 30526631 PMC6288901

[61] Narendra Varun, Rocha Pedro P, An Disi, Raviram Ramya, Skok Jane A, Mazzoni Esteban O, and Reinberg Danny. CTCF establishes discrete functional chromatin domains at the hox clusters during differentiation. *Science*, 347(6225):1017–1021, 2 2015 10.1126/science.1262088 25722416 PMC4428148

[62] Sandmann Thomas, Jakobsen Janus S, and Furlong Eileen E M. ChIP-on-chip protocol for genome-wide analysis of transcription factor binding in *Drosophila melanogaster* embryos. *Nat. Protoc.*, 1(6):2839–2855, 2006 10.1038/nprot.2006.383 17406543

[63] Ramani Vijay, Cusanovich Darren A, Hause Ronald J, Ma Wenxiu, Qiu Ruolan, Deng Xinxian, Blau C Anthony, Disteche Christine M, Noble William S, Shendure Jay, and Duan Zhijun. Mapping 3D genome architecture through *in situ* DNase Hi-C. *Nat. Protoc.*, 11(11):2104–2121, 11 2016 10.1038/nprot.2016.126 27685100 PMC5547819

[64] Mackay Trudy F C, Richards Stephen, Stone Eric A, Barbadilla Antonio, Ayroles Julien F, Zhu Dianhui, Casillas Sònia, Han Yi, Magwire Michael M, Cridland Julie M, Richardson Mark F, Anholt Robert R H, Barrón Maite, Bess Crystal, Blankenburg Kerstin Petra, Carbone Mary Anna, Castellano David, Chaboub Lesley, Duncan Laura, Harris Zeke, Javaid Mehwish, Jayaseelan Joy Christina, Jhangiani Shalini N, Jordan Katherine W, Lara Fremiet, Lawrence Faye, Lee Sandra L, Librado Pablo, Linheiro Raquel S, Lyman Richard F, Mackey Aaron J, Munidasa Mala, Muzny Donna Marie, Nazareth Lynne, Newsham Irene, Perales Lora, Pu Ling-Ling, Qu Carson, Ràmia Miquel, Reid Jeffrey G, Rollmann Stephanie M, Rozas Julio, Saada Nehad, Turlapati Lavanya, Worley Kim C, Wu Yuan-Qing, Yamamoto Akihiko, Zhu Yiming, Bergman Casey M, Thornton Kevin R, Mittelman David, and Gibbs Richard A. The *Drosophila melanogaster* genetic reference panel. *Nature*, 482(7384):173–178, 2 2012 10.1038/nature10811 22318601 PMC3683990

[65] Langmead Ben and Salzberg Steven L. Fast gapped-read alignment with bowtie 2. *Nat. Methods*, 9(4):357–359, 3 2012 10.1038/nmeth.1923 22388286 PMC3322381

[66] Durand Neva C, Robinson James T, Shamim Muhammad S, Machol Ido, Mesirov Jill P, Lander Eric S, and Aiden Erez Lieberman. Juicebox provides a visualization system for Hi-C contact maps with unlimited zoom. *Cell Syst*, 3(1):99–101, 7 2016 10.1016/j.cels.2015.07.012 27467250 PMC5596920

[67] Thurmond Jim, Goodman Joshua L, Strelets Victor B, Attrill Helen, Gramates L Sian, Marygold Steven J, Matthews Beverley B, Millburn Gillian, Antonazzo Giulia, Trovisco Vitor, Kaufman Thomas C, Calvi Brian R, and Consortium FlyBase. FlyBase 2.0: the next generation. *Nucleic Acids Res.*, 47(D1):D759–D765, 1 2019 10.1093/nar/gky1003 30364959 PMC6323960

[68] Kim Daehwan, Paggi Joseph M, Park Chanhee, Bennett Christopher, and Salzberg Steven L. Graph-based genome alignment and genotyping with HISAT2 and HISAT-genotype. *Nat. Biotechnol.*, 37(8):907–915, 8 2019 10.1038/s41587-019-0201-4 31375807 PMC7605509

[69] Pertea Mihaela, Pertea Geo M, Antonescu Corina M, Chang Tsung-Cheng, Mendell Joshua T, and Salzberg Steven L. StringTie enables improved reconstruction of a transcriptome from RNA-seq reads. *Nat. Biotechnol.*, 33(3):290–295, 3 2015 10.1038/nbt.3122 25690850 PMC4643835

[70] Bolger Anthony M, Lohse Marc, and Usadel Bjoern. Trimmomatic: a flexible trimmer for illumina sequence data. *Bioinformatics*, 30(15):2114–2120, 8 2014 10.1093/bioinformatics/btu170 24695404 PMC4103590

[71] Li Heng and Durbin Richard. Fast and accurate short read alignment with Burrows-Wheeler transform. *Bioinformatics*, 25(14):1754–1760, 7 2009 10.1093/bioinformatics/btp324 19451168 PMC2705234

[72] Quinlan Aaron R. BEDTools: The Swiss-Army tool for genome feature analysis. *Curr. Protoc. Bioinformatics*, 47:11.12.1–34, 9 2014 10.1002/0471250953.bi1112s47 25199790 PMC4213956

[73] A F A Smit, R Hubley, and P Green. RepeatMasker open, 2013.

[74] Fornes Oriol, Castro-Mondragon Jaime A, Khan Aziz, Lee Robin van der, Zhang Xi, Richmond Phillip A, Modi Bhavi P, Correard Solenne, Gheorghe Marius, Baranašić Damir, Santana-Garcia Walter, Tan Ge, Chèneby Jeanne, Ballester Benoit, Parcy François, Sandelin Albin, Lenhard Boris, Wasserman Wyeth W, and Mathelier Anthony. JASPAR 2020: update of the open-access database of transcription factor binding profiles. *Nucleic Acids Res.*, 48(D1):D87–D92, 1 2020 10.1093/nar/gkz1001 31701148 PMC7145627

[75] Bailey Timothy L, Boden Mikael, Buske Fabian A, Frith Martin, Grant Charles E, Clementi Luca, Ren Jingyuan, Li Wilfred W, and Noble William S. MEME SUITE: tools for motif discovery and searching. *Nucleic Acids Res.*, 37:W202–8, 7 2009 10.1093/nar/gkp335 19458158 PMC2703892

[76] Simon Anders, Paul Theodor Pyl, and Wolfgang Huber. HTSeq—a python framework to work with high-throughput sequencing data. August 2014. 10.1093/bioinformatics/btu638 PMC4287950 25260700

[77] Gurudatta B V, Yang Jingping, Van Bortle Kevin, Donlin-Asp Paul G, and Corces Victor G. Dynamic changes in the genomic localization of DNA replication-related element binding factor during the cell cycle. *Cell Cycle*, 12(10):1605–1615, 5 2013 10.4161/cc.24742 23624840 PMC3680540

