# Supplementary figures and images for: 3D genome evolution and reorganization in the Drosophila melanogaster species group
Source: PLoS Genet. 2020 Dec 7;16(12):e1009229. doi: 10.1371/journal.pgen.1009229 (PMC7746282; doi:10.1371/journal.pgen.1009229)

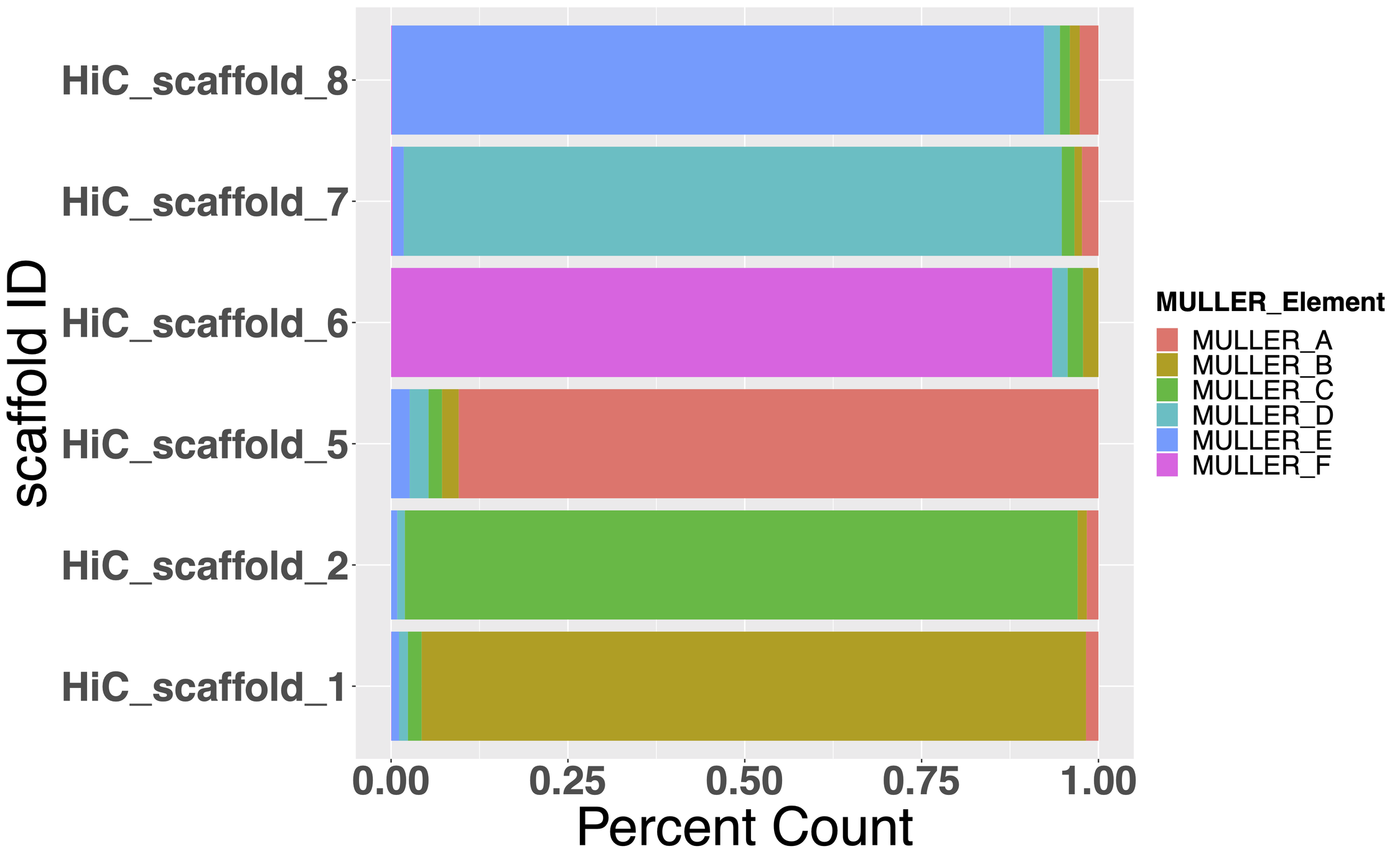

Supplement: S1 Fig — Percent of D. melanogaster genes corresponding to each of the D. triauraria chromosome-length scaffolds. Each scaffold is enriched for genes belonging to a single Muller element. (TIF) [file pgen.1009229.s001.tif]

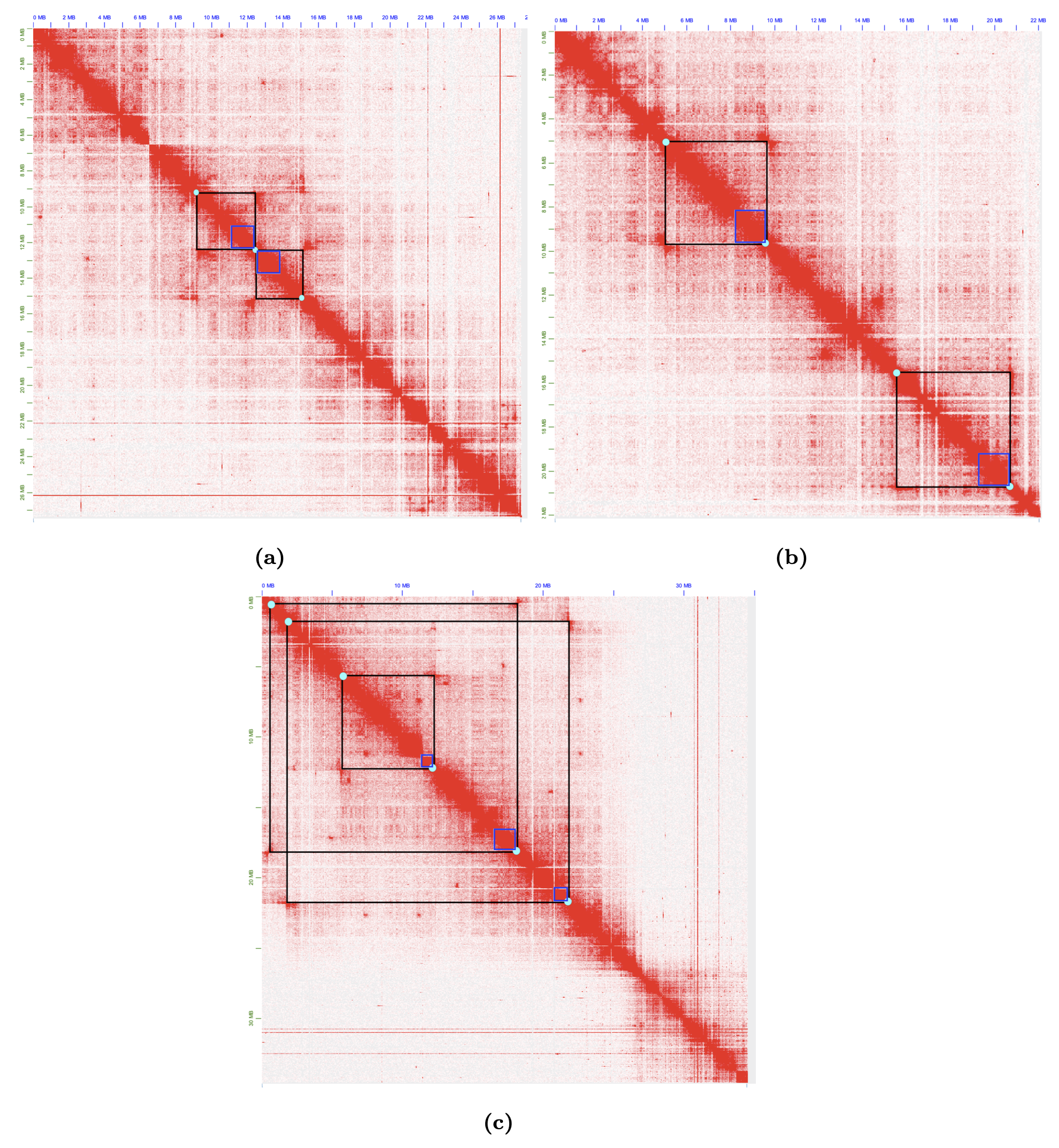

Supplement: S2 Fig — A) Muller B B) Muller C and C) Muller D. Light blue dots represent inversion breakpoints, black lines outline entire inverted sections. Dark blue rectangles highlight high contact frequencies along the diagonal demonstrating that inversions are polymorphic. (TIF) [file pgen.1009229.s002.tif]

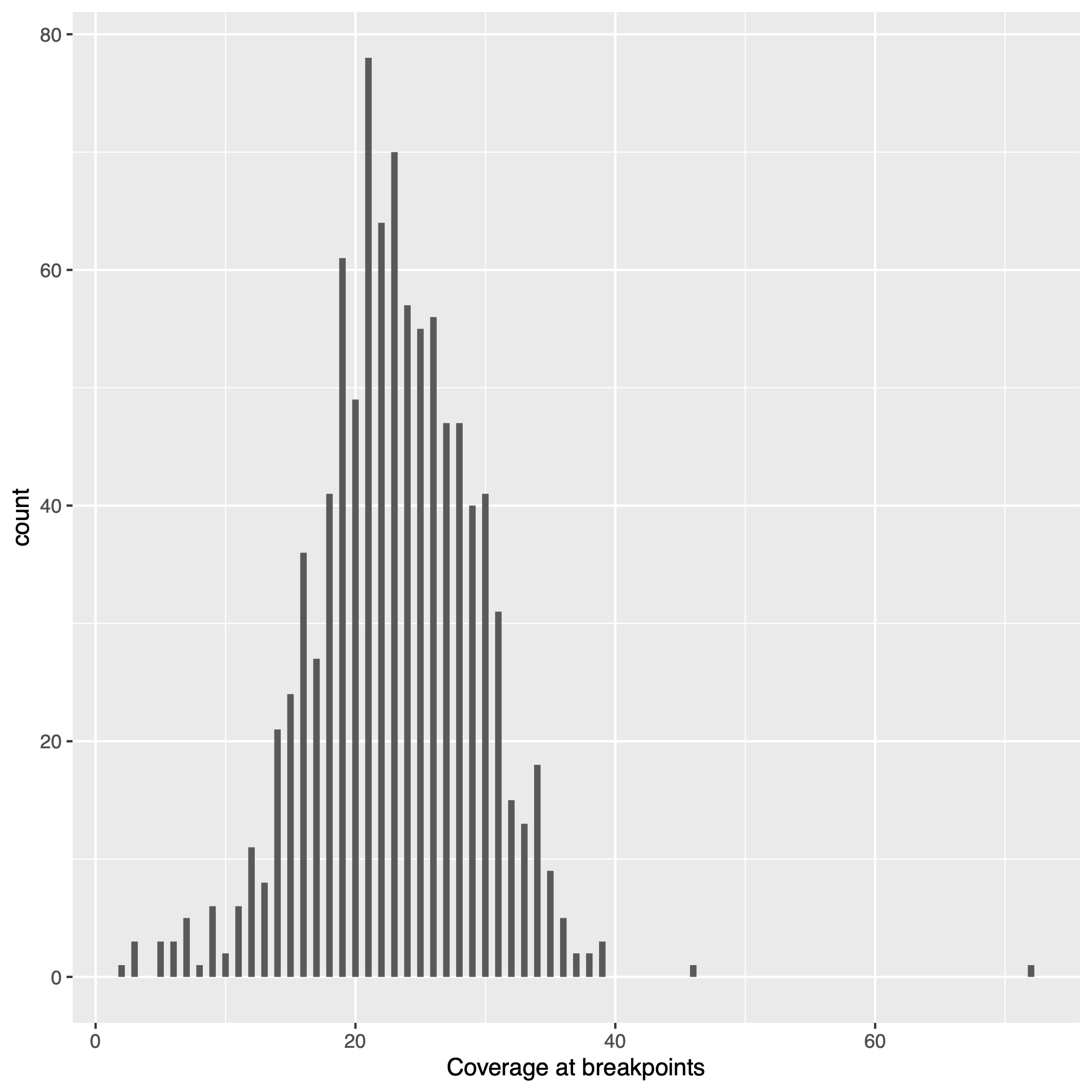

Supplement: S3 Fig — (TIF) [file pgen.1009229.s003.tif]

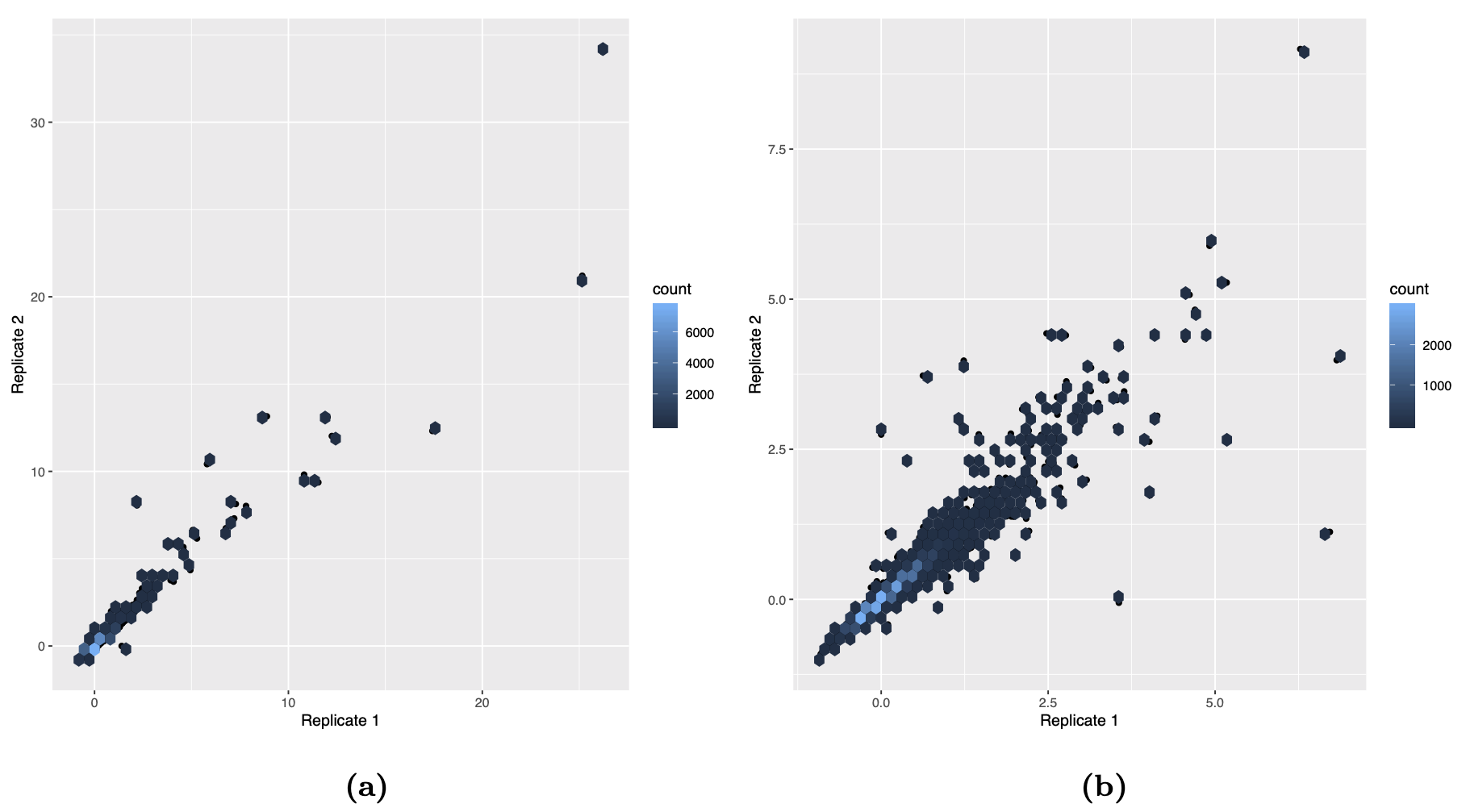

Supplement: S4 Fig — TAD separation scores from each replicate plotted for A) D. melanogaster (Spearman’s rho: 0.995) and B) D. triauraria (Spearman’s rho: 0.990). (TIF) [file pgen.1009229.s004.tif]

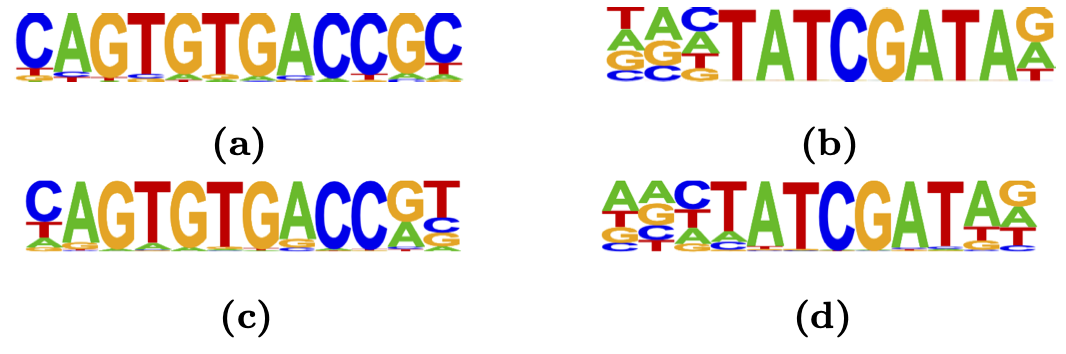

Supplement: S5 Fig — Motifs for A) D. melanogaster M1BP (p = 1e–17), B) D. melanogaster BEAF-32/DREF (p = 1e-18), C) D. triauraria M1BP (p = 1e–42), and D) D. triauraria BEAF-32/DREF (p = 1e–15). Homer [49] software found these sequence motifs to be enriched at TAD boundaries in D. melanogster and D. triauraria. BEAF-32 and DREF binding motifs are almost identical and both are recognized by the BEAF-32 insulator protein [77]. (TIF) [file pgen.1009229.s005.tif]

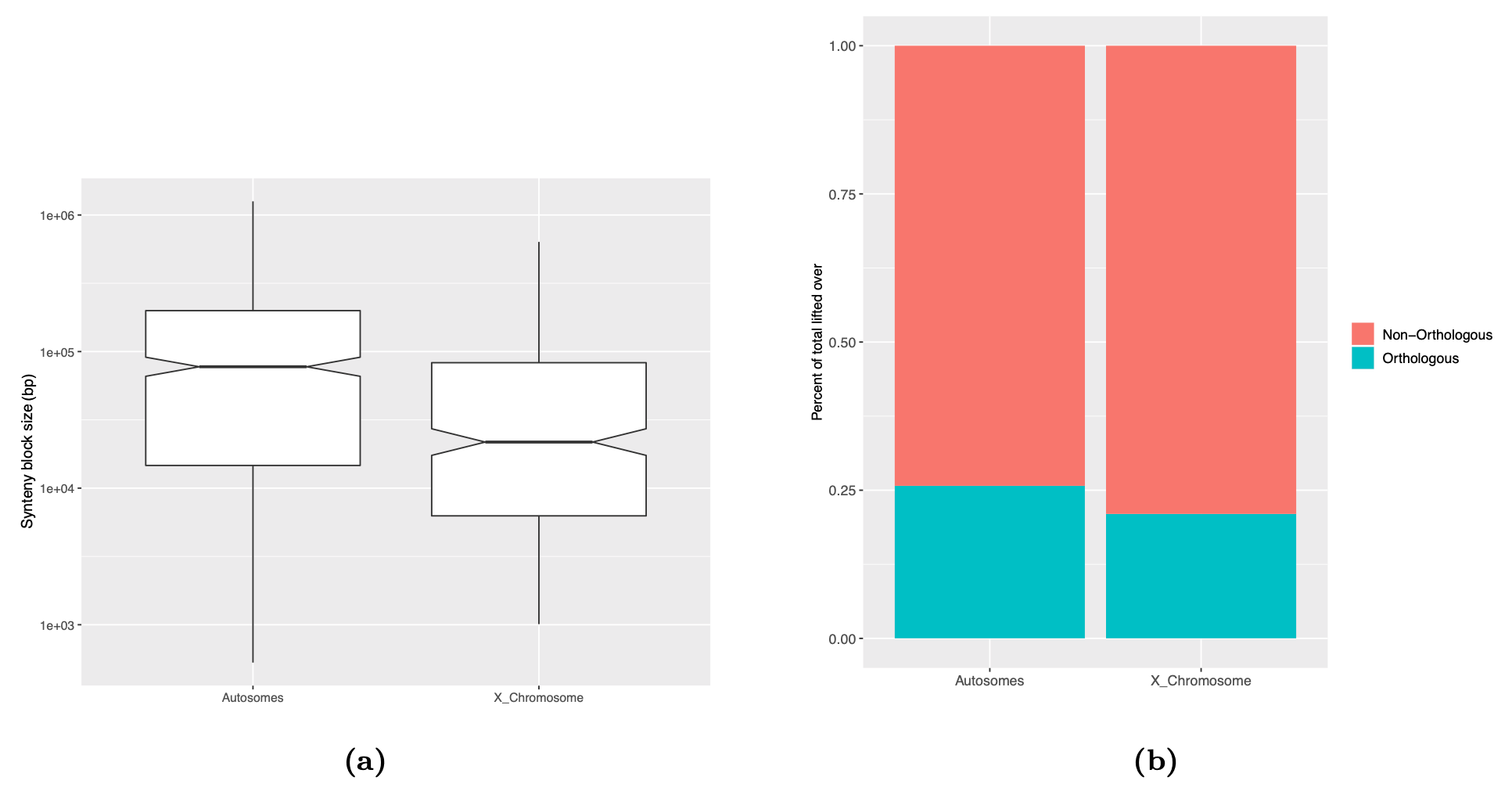

Supplement: S6 Fig — (A) The size of D. melanogaster synteny blocks is significantly reduced on the X chromosome (Muller A) relative to the autosomes, indicating that the X has accumulated more chromosomal rearrangements (Wilcoxon test p = 6.7e–05). (B) The proportion of orthologous lifted-over TAD domains is also significantly reduced on the X chromosome (Fisher’s Exact Test p = 0.0135). (TIF) [file pgen.1009229.s006.tif]

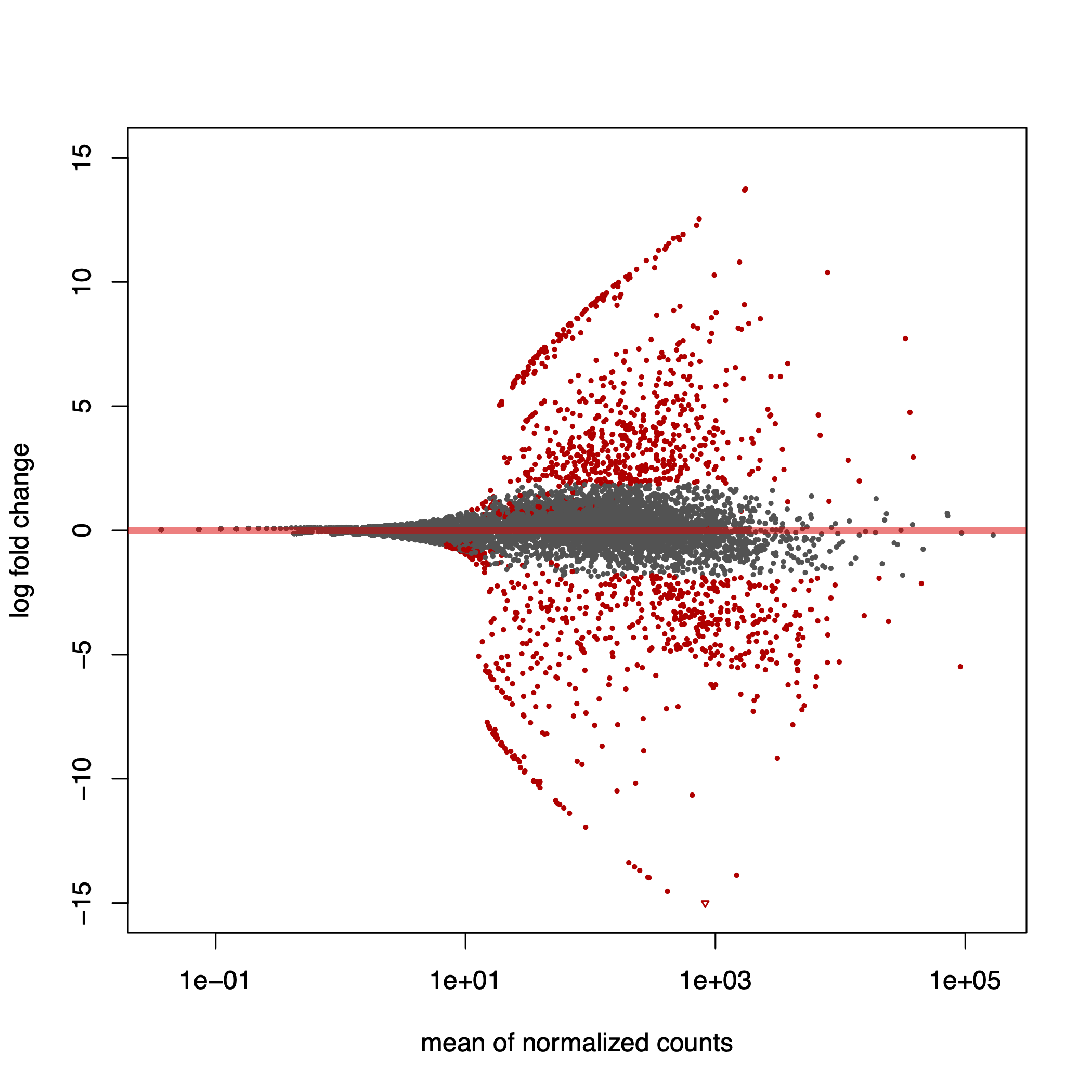

Supplement: S7 Fig — Differentially-expressed genes (adjusted p-value <= 0.05) between D. melanogaster and D. triauraria identified by DEseq2 [52] are shown in red. Each point represents an orthologous gene pair between the two species. The plot was created using the DEseq2 shrunken log2 fold changes which removes noise from low count genes. (TIF) [file pgen.1009229.s007.tif]

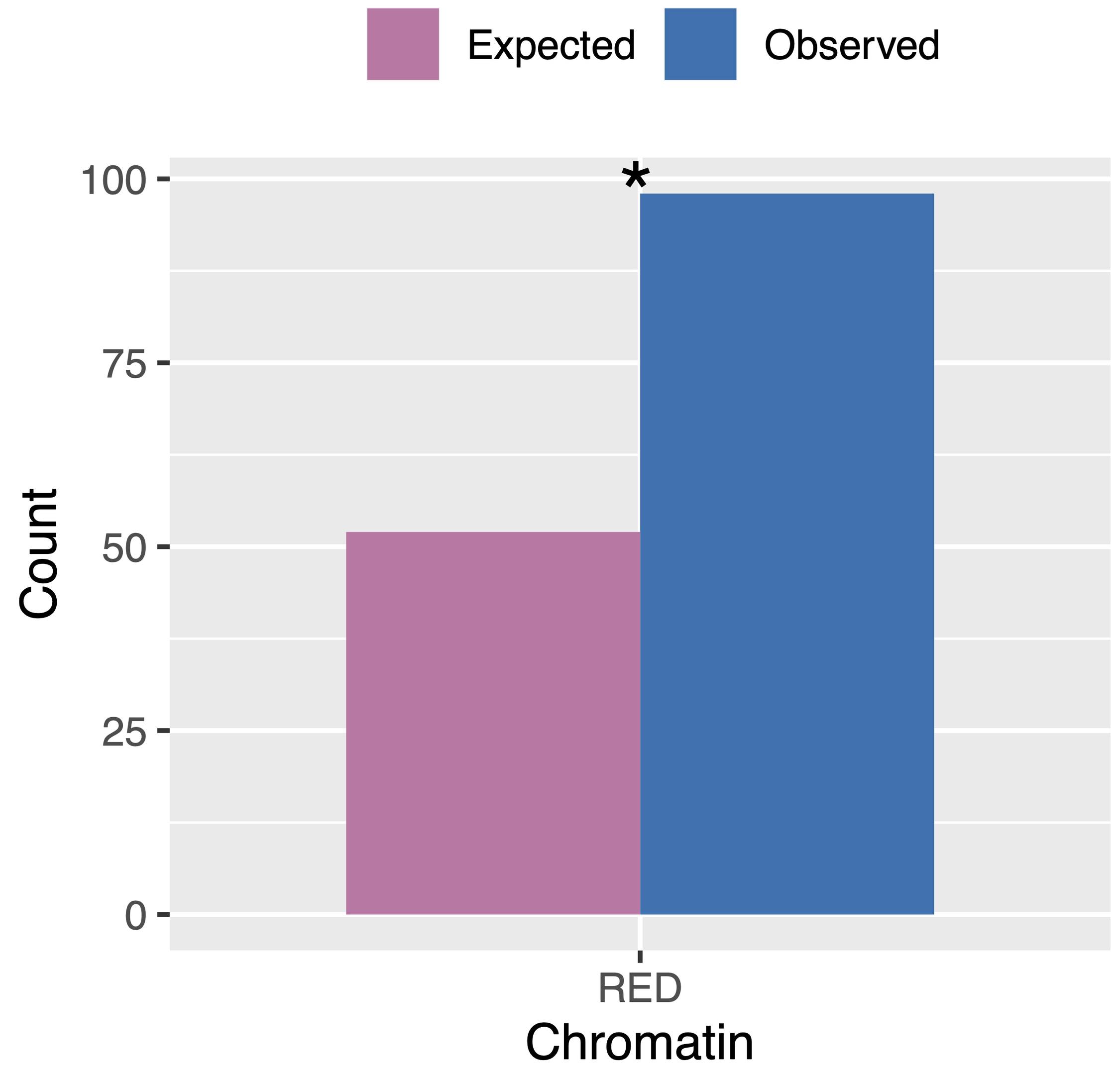

Supplement: S8 Fig — Permutation p < 0.001. (TIF) [file pgen.1009229.s008.tif]

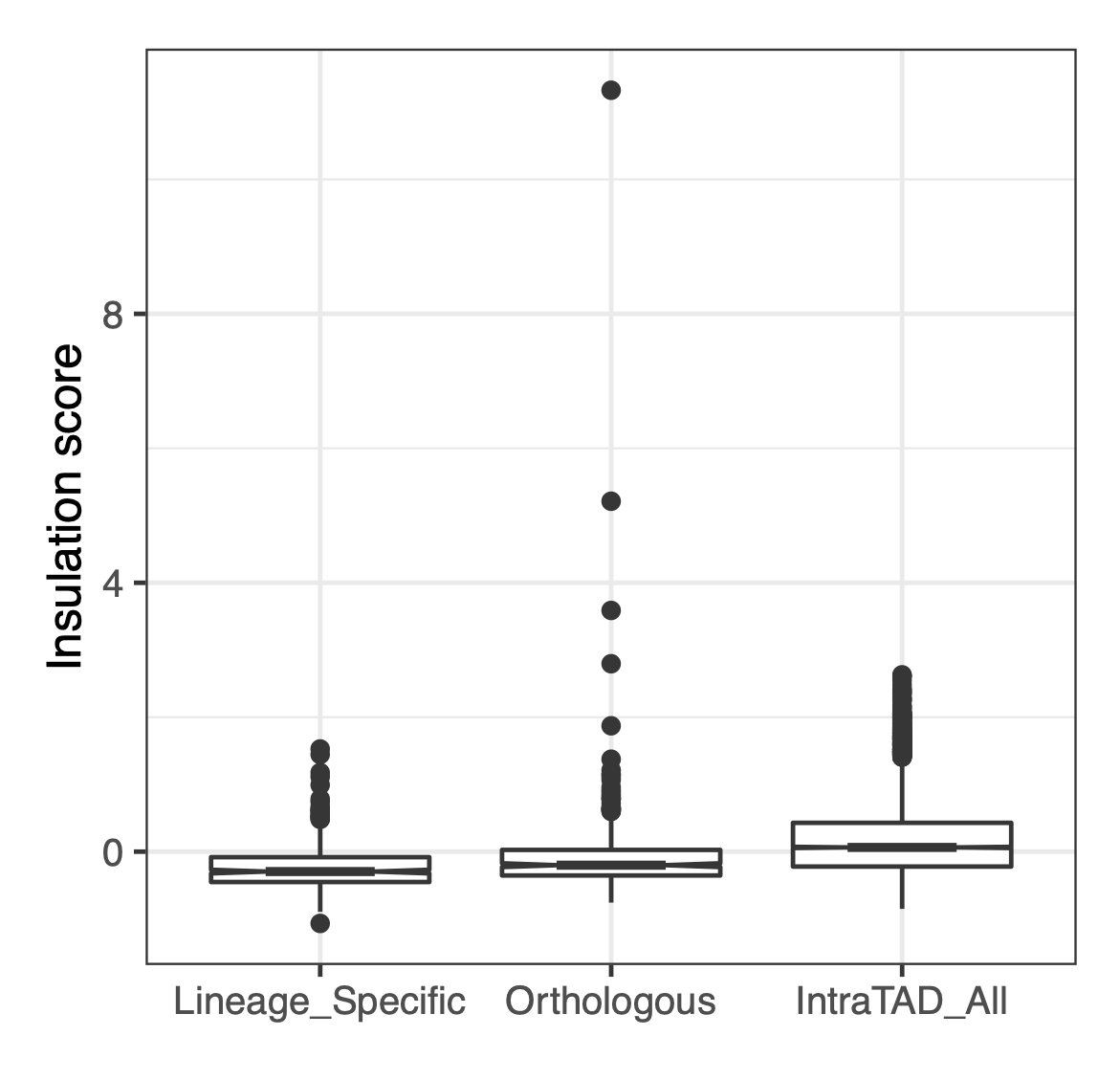

Supplement: S9 Fig — Outlier points were removed from Fig 6B to aid in visualization. All datapoints are shown here. (TIF) [file pgen.1009229.s009.tif]
