# Supplementary material for: 3D genome evolution and reorganization in the Drosophila melanogaster species group
Source: PLoS Genet. 2020 Dec 7;16(12):e1009229. doi: 10.1371/journal.pgen.1009229 (PMC7746282; doi:10.1371/journal.pgen.1009229)
Supplement: S1 Table — This study and Miller et al. [34]. The decrease in assembly size after scaffolding is mainly due to the removal of contigs by Purge haplotigs [38]. (PDF) [file pgen.1009229.s010.pdf]

	**Nanopore Sequencing (this study)**	**Nanopore Sequencing (combined)**	**200M Genome Assembly**	**Post Quality Control and Hi-C Scaffolding**
Number of Sequences/Contigs	633,844	1,043,600	1,098	302
Sum of Sequence/Contig Length	6,514,819,227	10,462,257,773	268,538,400	198,798,533
Average Sequence/Contig Length	10,278	10,025	244,570.49	658,273
Median Sequence Length	7,405	6,549	60,582.50	24,380
Longest Sequence Length	123,032	238,837	7,592,683	37,891,417
N50	–	–	1,274,151	34,538,105
