# Supplementary material for: 3D genome evolution and reorganization in the Drosophila melanogaster species group
Source: PLoS Genet. 2020 Dec 7;16(12):e1009229. doi: 10.1371/journal.pgen.1009229 (PMC7746282; doi:10.1371/journal.pgen.1009229)
Supplement: S2 Table — (PDF) [file pgen.1009229.s011.pdf]

Species	Replicate	Total number of read pairs	Pairs mappable, unique and high quality	Pairs used
D. melanogaster	1	126758371	65470274	41063921
D. melanogaster	2	157512176	71652238	45294778
D. triauraria	1	56128496	18491258	14551364
D. triauraria	2	57584713	19513854	15023262
