# Supplementary material for: 3D genome evolution and reorganization in the Drosophila melanogaster species group
Source: PLoS Genet. 2020 Dec 7;16(12):e1009229. doi: 10.1371/journal.pgen.1009229 (PMC7746282; doi:10.1371/journal.pgen.1009229)
Supplement: S3 Table — (PDF) [file pgen.1009229.s012.pdf]

**Species**	**Boundaries**		**Domains**	
	**High Confidence**	**Low Confidence**	**High Confidence**	**Low Confidence**
D. melanogaster	701	249	552	593
D. triauraria	843	355	639	811
