# Supplementary material for: 3D genome evolution and reorganization in the Drosophila melanogaster species group
Source: PLoS Genet. 2020 Dec 7;16(12):e1009229. doi: 10.1371/journal.pgen.1009229 (PMC7746282; doi:10.1371/journal.pgen.1009229)
Supplement: S4 Table — (PDF) [file pgen.1009229.s013.pdf]

**Category**	**Boundaries**	**Domains**
Total in *D. triauraria*	834	639
Unique lifted over to *D. melanogaster*	798	637
Orthologous	503	117
Non-orthologous	295	520
Non-orthologous (truncated/expanded)	–	73
Non-orthologous (split by lineage-specific boundary)	–	198
Non-orthologous (split by rearrangement)	–	249
Missing	36	2
