# Supplementary material for: 3D genome evolution and reorganization in the Drosophila melanogaster species group
Source: PLoS Genet. 2020 Dec 7;16(12):e1009229. doi: 10.1371/journal.pgen.1009229 (PMC7746282; doi:10.1371/journal.pgen.1009229)
Supplement: S5 Table — Paired Wilcoxon test p = 0.0059. (PDF) [file pgen.1009229.s014.pdf]

**Motif Comparison**	**Boundaries**
More	136
Fewer	95
Same	72
