# Supplementary material for: 3D genome evolution and reorganization in the Drosophila melanogaster species group
Source: PLoS Genet. 2020 Dec 7;16(12):e1009229. doi: 10.1371/journal.pgen.1009229 (PMC7746282; doi:10.1371/journal.pgen.1009229)
Supplement: S6 Table — (PDF) [file pgen.1009229.s015.pdf]

| **Chromatin State** | **Orthologous TADs: number of genes** | **Non-orthologous TADs: number of genes** |
|---|---|---|
| BLACK | 719 | 2156 |
| BLUE | 247 | 861 |
| GREEN | 20 | 357 |
| RED | 116 | 516 |
| YELLOW | 551 | 3373 |
